# Capsaicin-Induced Endocytosis of Endogenous Presynaptic Ca_V_2.2 in DRG-Spinal Cord Co-Cultures Inhibits Presynaptic Function

**DOI:** 10.1093/function/zqac058

**Published:** 2022-11-25

**Authors:** Krishma H Ramgoolam, Annette C Dolphin

**Affiliations:** Department of Neuroscience, Physiology and Pharmacology, University College London, London, WC1E 6BT, UK; Department of Neuroscience, Physiology and Pharmacology, University College London, London, WC1E 6BT, UK

**Keywords:** Ca_V_2.2 channels, calcium imaging, DRG neurons, TRPV1 channels, protein trafficking, rab11, capsaicin

## Abstract

The N-type calcium channel, Ca_V_2.2 is key to neurotransmission from the primary afferent terminals of dorsal root ganglion (DRG) neurons to their postsynaptic targets in the spinal cord. In this study, we have utilized Ca_V_2.2_HA knock-in mice, because the exofacial epitope tag in Ca_V_2.2_HA enables accurate detection and localization of endogenous Ca_V_2.2. Ca_V_2.2_HA knock-in mice were used as a source of DRGs to exclusively study the presynaptic expression of N-type calcium channels in co-cultures between DRG neurons and wild-type spinal cord neurons. Ca_V_2.2_HA is strongly expressed on the cell surface, particularly in TRPV1-positive small and medium DRG neurons. Super-resolution images of the presynaptic terminals revealed an increase in Ca_V_2.2_HA expression and increased association with the postsynaptic marker Homer over time in vitro. Brief application of the TRPV1 agonist, capsaicin, resulted in a significant down-regulation of cell surface Ca_V_2.2_HA expression in DRG neuron somata. At their presynaptic terminals, capsaicin caused a reduction in Ca_V_2.2_HA proximity to and co-localization with the active zone marker RIM 1/2, as well as a lower contribution of N-type channels to single action potential-mediated Ca^2+^ influx. The mechanism of this down-regulation of Ca_V_2.2_HA involves a Rab11a-dependent trafficking process, since dominant-negative Rab11a (S25N) occludes the effect of capsaicin on presynaptic Ca_V_2.2_HA expression, and also prevents the effect of capsaicin on action potential-induced Ca^2+^ influx. Taken together, these data suggest that capsaicin causes a decrease in cell surface Ca_V_2.2_HA expression in DRG terminals via a Rab11a-dependent endosomal trafficking pathway.

## Graphical Abstract

**Graphical Abstract:**
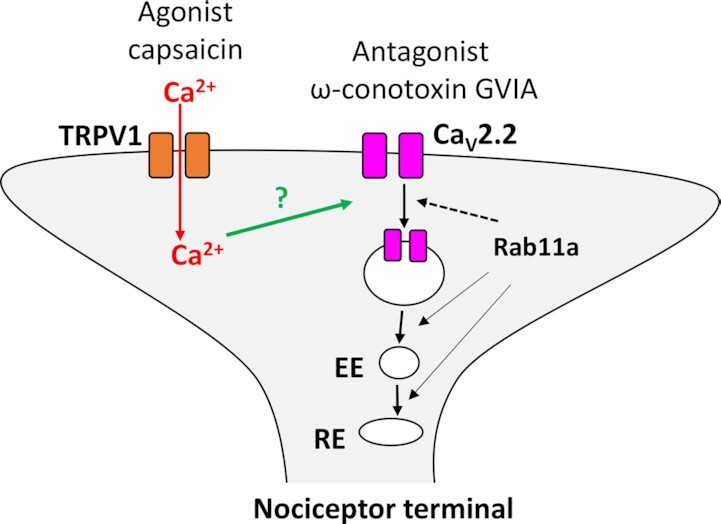

## Introduction

Primary afferent axons of peripheral sensory dorsal root ganglion (DRG) neurons synapse in the dorsal horn of the spinal cord. The N-type calcium channel, Ca_V_2.2, is the main presynaptic voltage-gated calcium (Ca_V_) channel (VGCC) expressed in DRG neurons.^1,2^ It is a key mediator of nociceptive transmission, and it is important as a therapeutic target.^3,4^

Ca_V_ channels are multisubunit complexes composed of the central pore-forming α1 and auxiliary subunits, α_2_δ and β. A significant role for the α_2_δ-1 isoform has been established in chronic neuropathic pain.^5–8^ Furthermore, the gabapentinoids, including gabapentin, which are used in neuropathic pain conditions, act on α_2_δ-1.^8,9^ The mechanism by which gabapentin reduces Ca^2+^ currents entails a reduction in trafficking of α_2_δ-1 and the associated calcium channel complex.^10^ This involves interference with recycling of α_2_δ-1 from Rab11a-positive recycling endosomes to the plasma membrane.^11,12^

TRPV1 is a nonselective cation channel, which is Ca^2+^-permeable and activated by the exogenous ligand capsaicin.^13^ It is located in distinct subpopulations of small and medium DRG neurons and plays an essential role in nociception, particularly noxious heat.^13–17^ Whereas brief exposure to capsaicin produces pain, it then desensitizes DRG neurons, resulting in reduced nociception,^18^ and a reduction in dorsal horn synaptic transmission.^19^ Ca^2+^ influx through TRPV1 channels initiates a cascade of events, one of them being changes in VGCC expression.^20^ However, the mechanisms behind the functional interaction between TRPV1 channels and VGCCs in primary nociceptors remains poorly understood.

Co-cultures between DRG and spinal cord neurons are an ideal model system for the study of synaptic transmission at primary afferent synapses.^21–24^ In previous studies, these co-cultures were also used to explore the mechanistic link between TRPV1 and Ca^2+^ signaling.^21^ It was demonstrated that a brief stimulation of TRPV1 with capsaicin resulted in glutamate release, independent of N-type calcium channels.^21^ It was also postulated that Ca^2+^ influx through TRPV1 mediated a Ca^2+^/calcineurin-dependent inhibition of N-type currents in DRG cell bodies.^20^

In the present study, we examine the maturation of the localization of Ca_V_2.2 both in DRG neuronal somata and at their presynaptic terminals over time, by using DRG neurons from Ca_V_2.2_HA^KI/KI^ mice,^25^ co-cultured with wild-type spinal cord neurons. We then use these co-cultures to examine the effects of capsaicin on the distribution and presynaptic function of Ca_V_2.2_HA. We sought to determine whether capsaicin changes the expression of cell surface Ca_V_2.2, and to understand the mechanisms influencing presynaptic localization and function of this channel. Our results reveal a dramatic effect of brief capsaicin exposure on Ca_V_2.2 distribution in the somata and in presynaptic terminals of DRG neurons, which occurs with a slow time course and involves a Rab11a-dependent pathway. Our results support the view that presynaptic Ca_V_2.2 channels are highly dynamic in their localization and activity-dependent turnover.

## Materials and Methods

### Animals

The Ca_V_2.2_HA^KI/KI^ C57BL/6 mouse line described previously^25^ and wild-type C57BL/6 mice were housed in groups of no more than five on a 12-h:12-h light:dark cycle; food and water were available ad libitum. Both Ca_V_2.2_HA^KI/KI^ and Ca_V_2.2^WT/WT^ mice were obtained by breeding from homozygotes. All experimental procedures were covered by UK Home Office licenses and had local ethical approval by the University College London (UCL) Bloomsbury Animal Welfare and Ethical Review Body. All cultures were prepared from P0–P1 male and female mice.

### DRG-Spinal Cord Neuronal Co-Cultures

Dorsal root ganglion neurons from Ca_V_2.2_HA^KI/KI^ mice were cultured with spinal cord neurons from Ca_V_2.2^WT/WT^ P0/1 mice. DRG and spinal cords were extracted in ice cold dissection medium (Leibovitz’s L15 Medium without supplements; Gibco). The spinal cord was dissected in small segments (∼0.5 mm) and then digested in 2.5% trypsin (Gibco)and 1200 U/µL DNase I (Sigma Aldrich) for 23 min at 37°C in a 5% CO_2_ incubator. The spinal cord sections were then washed with 37°C culture medium I (10% fetal bovine serum, 1 unit/mL penicillin, 1 μg/mL mg streptomycin and DMEM; Gibco). Following this, the spinal cord was triturated twice gently with fire polished glass pipettes. Spinal cord suspensions were plated onto either 22 or 25 mm glass coverslips, which were coated with poly-l-lysine and laminin.

Dorsal root ganglions were dissected and incubated in Hanks’ Balanced Salt solution (HBSS; Gibco^TM^) and kept at 4°C until all DRG tissues were removed. The DRGs were incubated in DRG enzyme solution (1000 U/mL DNAse I, 3.75 mg/mL dispase and 0.8 mg/mL collagenase type 1A; Gibco) for 21 min at 37°C in a 5% CO_2_ incubator. Culture medium I was added to the digested tissue, which was then transferred to a 1.5 mL Eppendorf tube and centrifuged in microfuge at 1000 rpm for 5 min. The supernatant was removed, the pellet was re-suspended in culture medium I and triturated three times with fire-polished glass pipettes to produce a single cell suspension. The cell suspension was centrifuged at 1000 rpm for 5 min and supernatant removed. Culture medium I was added to the pellet before the DRG neurons were plated on to the spinal cord neurons. The dishes were flooded with culture medium I (at 37°C), 1 h after plating. In this study, co-cultures used at DIV 1, 7, and 14 are referred to as immature cultures. Co-cultures utilized at DIV 21 and 28 are classified as mature cultures.

For experiments involving transfection, DRG neurons were transfected before plating. To ensure sufficient transfection of DRG neurons, DRG tissue was collected from three mice (P0/P1). After dissociation, the DRG cell suspension was washed with 1 mL of 37°C HBSS and centrifuged at 1000 rpm for 5 min. The supernatant was discarded, cell pellet was resuspended in 100 mL Nucleofector™ (Rat Neuron Nucleofector kit, Lonza) transfection reagent and electroporated with 2 μg of cDNA mix according to the manufacturer’s protocol. The following mixes were transfected separately for the experiments in this study: synaptophysin-GCaMP6f (Sy-GCaMP6f) and VAMP-mOrange 2 (VAMP-mOr2;^26^) (3:1); empty vector and mCherry (1:1); Rab11a (S25N) and mCherry (1:1); Empty Vector and mCherry (1:1); Rab11a (S25N), Sy-GCaMP6f, VAMP-mOr2 (1:1:1), and Empty Vector, Sy-GCaMP6f, VAMP-mOr2 (1:1:1). For expression in DRG neurons, Rab11a (S25N)^12^ was subcloned into the vector pCAGGs. Electroporated cells were then incubated in one 0% FBS-RPMI (Roswell Park Memorial Institute medium) for 5 min at 37°C in a 5% CO_2_ incubator. The DRG cell suspension was finally added dropwise on top of the plated spinal cord neurons.

For both electroporated and nonelectroporated cells, culture medium I was added to the cells 1 h after plating. The following day, culture medium I was replaced with culture medium II (DMEM supplemented with 24 μg/mL insulin (Sigma Aldrich), 100 μg/mL transferrin (EMD Millipore), 5% horse serum, 2× B27 supplement (Gibco), 1× GlutaMAX™, 0.5 ng/mL NGF (Sigma Aldrich), and 1 unit/mL penicillin, 1 μg/mL streptomycin). Half of the growth medium was replaced every 3–4 d with fresh culture medium II. To inhibit the proliferation of nonneuronal cells, after 48 h, 5 μm cytosine-a-d-arabinofuranoside (AraC; Gibco) was added to cultures for 12 h. Two days later, cells were again treated with 5 μm AraC for 12 h.

### Immunolabeling of DRG—Spinal Cord Neuronal Co-Cultures

All immunocytochemistry protocols included the use of blocking buffer (20% horse serum in PBS), antibodies were diluted in antibody solution (10% horse serum in PBS), and for permeabilized conditions 0.1%–0.3% v/v Triton X-100 in PBS was included. All co-cultures were fixed and postfixed [before and after incubation with primary rat anti-HA antibody (Roche), respectively] using 4% paraformaldehyde (PFA) and 4% sucrose in PBS for 5 min at room temperature (20°C). All primary and secondary antibodies were applied separately and consecutively to one another to prevent problems with cross-reactivity of antibodies.

#### Cell Surface Ca_V_2.2_HA in DRG Cell Bodies

Co-cultures were fixed and blocked for a minimum of 1 h at 20°C. To prevent nonspecific binding of secondary antibodies, cultures were first incubated with rat anti-HA (1:100) antibody overnight at 4°C followed by donkey antirat Alexa Fluor (AF) 488 (1:500; Invitrogen) for 1.5 h at 20°C before staining for other markers. Unbound primary antibody was washed off using PBS and cells were postfixed.

#### Ca_V_2.2_HA, Homer, and Vglut2

Co-cultures were fixed and blocked for 1 h at 20°C. First, for Ca_V_2.2_HA immunolabeling, cultures were incubated in antibodies as described above. Next, cultures were permeabilized by incubation in antibody solution containing 0.3% Triton X-100. Cultures were incubated with rabbit anti-Homer antibody (1:2000; Frontier Institute) antibody overnight at 4°C. Subsequently, cultures were incubated with donkey antirabbit AF 633 antibody (1:500; Invitrogen) for 1.5 h at 20°C. Finally, for vGluT2 immunolabeling, neurons were incubated with guinea pig (GP) anti-vGluT2 (1:5000; Merk Millipore) antibody overnight at 4°C. The next day, secondary donkey anti-GP AF 594 (1:500; Invitrogen) antibody was applied to neurons for 1.5 h at 20°C.

#### Ca_V_2.2_HA and RIM 1/2

Co-cultures were fixed and blocked for 1 h at 20°C. First, for Ca_V_2.2_HA immunolabeling, cultures were incubated using the protocol above. Following this, neurons were incubated with rabbit-anti-RIM 1/2 (1:200; Synaptic Systems) antibody overnight at 4°C. Next, secondary donkey anti-rabbit AF 594 (1:500; Invitrogen) antibody was applied to neurons for 1.5 h at 20°C.

#### Capsaicin Application and Immunocytochemical Protocol

Capsaicin at a concentration of 1 µm diluted in Krebs–Ringer–Hepes (KRH) was applied to co-cultured neurons at 21 DIV for 2 min at 37°C. For controls, neurons were incubated similarly in KRH but without capsaicin. After incubation with capsaicin, cultures were briefly washed and incubated in culture medium II for a 0-, 20-, 40-, or 60-min rest period at 37°C. Co-cultures were then fixed at 20°C for 5 min. Following this, cells were blocked for 1 h at 20°C.

#### Ca_V_2.2_HA and TRPV1

Co-cultures were fixed and blocked for 1 h at 20°C. First, for Ca_V_2.2_HA immunolabeling, cultures were incubated in antibodies as described above. After this, for TRPV1 immunolabeling, neurons were incubated with goat anti-TRPV1 (1:500; Santa Biotech) antibody overnight at 4°C in permeabilized conditions (0.1% Triton X-100 in antibody solution), followed by donkey anti-goat AF488 antibody (1:500; Invitrogen) for 1.5 h at 20°C.

#### Ca_V_2.2_HA, TRPV1, and RIM 1/2

For cell surface Ca_V_2.2_HA and TRPV1 labeling, the protocol above was used. Following this, cultures were incubated in rabbit anti-RIM 1/2 antibody (1:200) followed by donkey anti-rabbit AF647 antibody (1:500; Invitrogen).

#### Ca_V_2.2_HA, RIM 1/2, and Mcherry

For Ca_V_2.2_HA and RIM 1/2 immunolabeling, the protocols above were used. Subsequently, co-cultures were incubated in GP antired fluorescent protein (RFP) antibody at 1:500 (Synaptic Systems) overnight at 4°C. Next, secondary donkey anti-GP AF 594 antibody was applied to coverslips at 1:500 (Invitrogen) for 1.5 h at 20°C.

Following all immunocytochemical protocols, nuclei were stained with DAPI (4′,6-diamidino-2-phenylindole) (500 n m) before mounting on slides using Vectashield (Vector Laboratories) to reduce photobleaching.

### Image Acquisition and Analysis

Co-cultures were examined using super resolution Airyscan mode imaging on an LSM 780 confocal microscope (Zeiss) with ×63 objective (1768 × 1768 pixels) as z-stacks (0.173 μm optical section). Super-resolution images then underwent pixel reassignment and Airyscan processing (6×) using Zen software. Images were acquired with constant settings in each experiment from at least three separate cultures per experiment.

To quantify Ca_V_2.2_HA density and its association with vGlut2, Homer, and RIM 1/2, the Image J plugin: Distance Analysis (DiAna)^27^ was used. DiAna was used to quantify the distance measurements between centres of co-localized objects, percentages of co-localizing volumes for each object’s pair and mean intensity of puncta.

For cell body analysis, using ImageJ software (Schneider et al. 2012), every DRG neuron with a visible nucleus with Ca_V_2.2_HA immunolabeling was measured.^25^ A 10-pixel wide line (0.9 μm) was drawn following the perimeter of the cell from which the perimeter length was recorded as an estimation of the size of the cell (small <61 μm, medium 61–94 μm, or large >94 μm^28^); and the mean membrane HA fluorescence intensity.

### Live Cell Ca^2+^ Imaging

#### Ca^2+^ Imaging of Co-Cultures

Live cell Ca^2+^ imaging was performed at DIV 7, 14, 21, and 28, as previously described with minor modifications.^26,29^ Coverslips were mounted in a rapid-switching, laminar-flow perfusion, and stimulation chamber (RC-21BRFS, Warner Instruments) on the stage of an epifluorescence microscope (Axiovert 200 M, Zeiss). Live cell images were acquired with an Andor iXon+ (model DU-897U-CS0-BV) back-illuminated EMCCD camera using OptoMorph software (Cairn Research, UK). White and 470 n m LEDs served as light sources (Cairn Research, UK). Fluorescence excitation and collection was done through a Zeiss 40 × 1.3 NA Fluor objective using 450/50 n m excitation and 510/50 n m emission and 480 n m dichroic filters (for sy-GCaMP6f) and a 545/25 n m excitation and 605/70 n m emission and 565 n m dichroic filters (for mOrange2). Action potentials (AP) were evoked by passing 1 ms current pulses via platinum electrodes. Cells were perfused (0.5 mL/min) in a saline solution at 25 °C containing (in mM) 119 NaCl, 2.5 KCl, 2 CaCl_2_, 2 MgCl_2_, 25 HEPES (buffered to pH 7.4), 30 glucose, 10 μm 6-cyano-7-nitroquinoxaline-2,3-dione (CNQX, Sigma) and 50 μm D, L-2-amino-5-phosphonovaleric acid (AP5, Sigma). Images were acquired at 100 Hz over a 512 × 266 pixel area in frame transfer mode (exposure time 7 ms) and analyzed in ImageJ (http://rsb.info.nih.gov/ij) using a custom-written plugin (http://rsb.info.nih.gov/ij/plugins/time-series.html). Successfully transfected neurons were identified by visualizing sy-GCaMP6f fluorescence in response to a 33 Hz stimulation for 180 ms every 4 s. Subsequently, single stimulations of 1 ms (mimicking a single AP) were repeated 5 times with 30 s intervals. Regions of interest (ROI, 2 μm diameter circles) were placed around synaptic boutons responding to an electrical stimulation of 10 AP at 60 Hz. Functional synaptic boutons were identified by the increase of fluorescence of VAMP-mOr2 in response to 200 AP stimulation at 10 Hz (in this case, images were acquired at 2 Hz with 50 ms exposure time). ω-conotoxin GVIA (1 μm; CTX, Alomone Labs) was perfused for at least 2 min. To determine if any of the observed reduction in fluorescence was due to bleaching, control experiments were performed where CTX was replaced with normal imaging medium and perfused for 2 min. Cells were then re-stimulated and a reduction of 6.9% ± 4.0% was recorded during 1 AP stimulation. All the values shown here have been adjusted for this reduction.

#### Ca^2+^ Imaging of Capsaicin Treated Co-Cultures Following Perfusion of Control Medium for 20–60-min Rest Period

An initial protocol consisting of 1 AP and 200 APs stimulations was applied to assess the baseline responses. After 5 min, either capsaicin or control medium was applied for 2 min. Control medium was then perfused for 20, 40, or 60 min. To assess the contribution of N-type calcium channels to the Ca^2+^ transient following 1AP, CTX was applied for 2 min. A final 1 AP train stimulation was applied to the cultures and the initial Ca^2+^ transients were used to normalize the final Ca^2+^ transient to determine the contribution of N-type VGCCs. To determine if any of the observed reduction in fluorescence of capsaicin-treated or control-treated neurons was due to bleaching, control experiments were performed where CTX was replaced with normal imaging medium and perfused for 2 min. Following this, cells were perfused in normal live imaging medium for 20, 40, or 60 min. Control-treated cells were then re-stimulated with 1 AP and a reduction of 4.8% ± 7.0%, 11% ± 3.0%, and 5% ± 2% was recorded for cells perfused for 20, 40, or 60 min, respectively. Capsaicin-treated cells were also re-stimulated with 1 AP and a reduction of 0.03% ± 0.01%, 0.3% ± 0.03%, and 1.8% ± 0.6% was recorded for cells perfused for 20, 40, or 60 min, respectively. Values shown in this study have been adjusted for these reductions.

### Statistical Analysis

Data were analyzed with Prism 9.0 (GraphPad Software). Where error bars are shown, they are SEM; “*n*” refers to the number of separate cultures used (termed experiments), unless indicated otherwise. Statistical significance between two groups was assessed by Student’s *t*-test, as stated. One-way ANOVA and stated post-hoc analysis, recommended as appropriate by Prism, were used for comparison of means between three or more groups.

## Results

### Ca_V_2.2_HA Expression in DRG—Spinal Cord Neuron Co-Cultures

**Figure 1.**
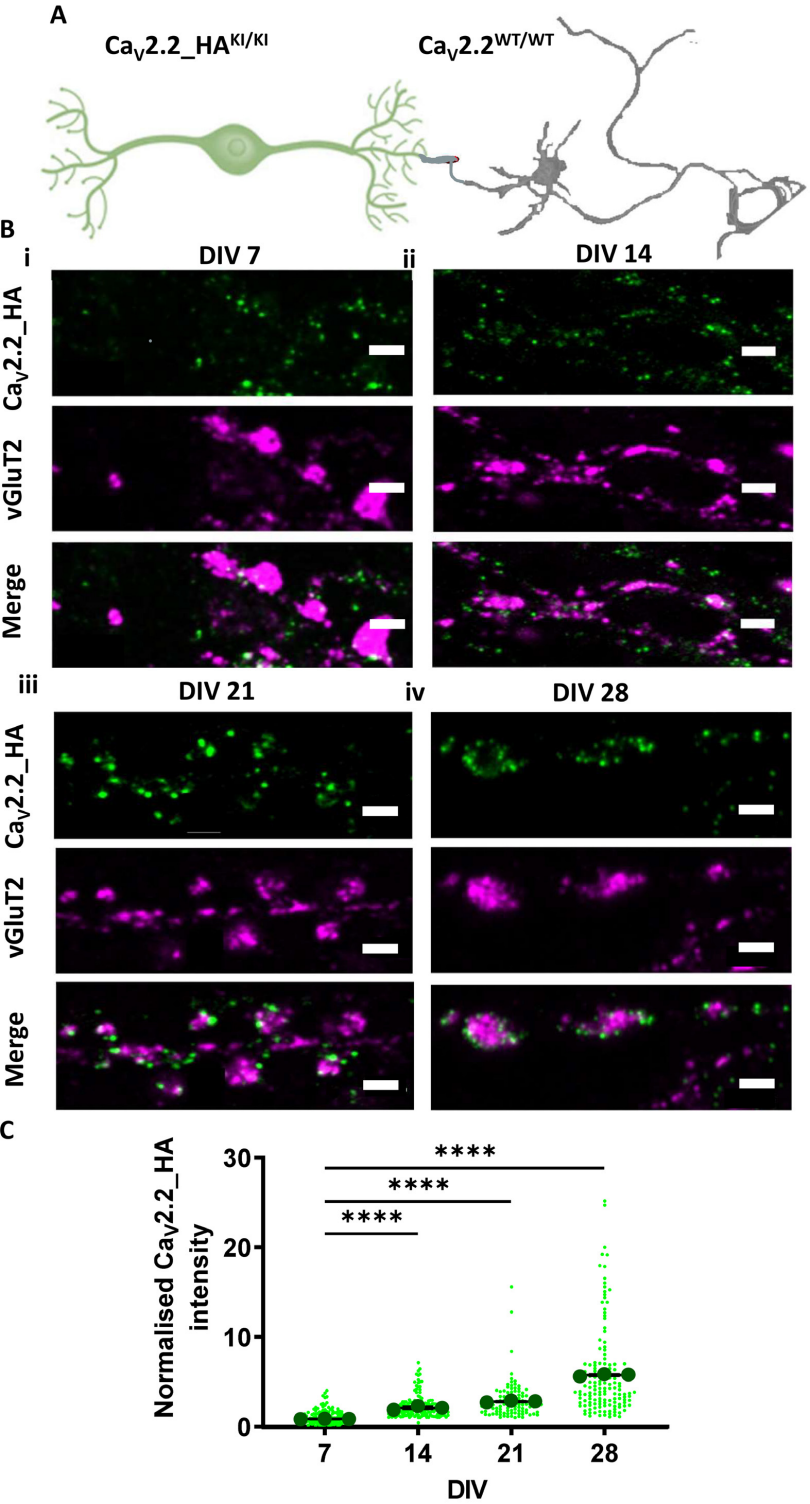
The development of Ca_V_2.2_HA presynaptic expression in DRG-spinal cord neuron co-cultures. (A) Schematic diagram of co-cultures composed of DRG neurons from Ca_V_2.2_HA^KI/KI^ mice (green) cultured with spinal cord neurons from Ca_V_2.2^WT/WT^ mice (gray). (B) Airyscan images of co-cultures at (i) DIV 7, (ii) DIV 14, (iii) DIV 21, and (iv) DIV 28. Ca_V_2.2_HA (top panel; green), vGluT2 (middle panel; magenta), and merged image (bottom panel). Scale bars: 2 μm. (C) Ca_V_2.2_HA intensity measured at presynaptic terminals of DRG neurons from co-cultures fixed at DIV 7, 14, 21, and 28 (green points). Individual data points represent 150 vGlut2 positive puncta also positive for Ca_V_2.2_HA. Mean ± SEM of the three experiments (black circles) are superimposed. Mean Ca_V_2.2_HA intensity of each experiment was normalized to that at DIV 7. Statistical analysis: one-way ANOVA with Sidak’s multiple selected comparison post-hoc test; ****P < .0001.

In the present study, a co-culture system was devised to recapitulate major characteristics of peripheral neuron maturation, synapse formation, and function. We combined DRG neurons cultured from Ca_V_2.2_HA^KI/KI^ mice with spinal cord neurons from Ca_V_2.2^WT/WT^ mice (Figure 1A). This ensured that the changes in Ca_V_2.2_HA being investigated were only those occurring in the presynaptic DRG neurons and their terminals. Dorsal root ganglion glial cells and spinal cord astrocytes served as substrates for the co-cultured neurons. Dorsal root ganglion neurites have shown a preferential growth in vitro into dorsal spinal cord explants.^30^ The time course for functional synapse formation between DRG neurons with their dorsal horn partners has been reported to commence at day in vitro (DIV) 5,^31^ and previous studies have investigated these processes in co-cultures for up to 4 wk.^22,23^

The clustering of synaptic vesicles at presynaptic active zones is one of the initial signs of synapse formation. All primary afferents use glutamate as their major neurotransmitter, thus having an excitatory effect on their postsynaptic targets.^32,33^ Spinal cord synaptic vesicles are enriched in vesicular glutamate transporters (vGluT)-1 and vGluT2.^34,35^ vGluT2 immunolabeling has been shown to be most prominent in Lamina II of the spinal cord, suggesting that it is associated with glutamatergic transmission from small diameter nociceptors.^34^ For this reason, vGluT2 was used here as a presynaptic marker to identify glutamatergic synapses.

Ca_V_2.2_HA expression was examined in co-cultures at different timepoints: DIV 7, 14, 21, and 28 (Figure 1B and C). At DIV 7, Ca_V_2.2_HA puncta were observed diffusely throughout the neurites, and were not strongly associated with vGlut2 puncta (Figure 1Bi and C). In contrast, in DIV 28 co-cultures, both Ca_V_2.2_HA and vGlut2 showed a strong punctate staining profile along the processes (Figure 1 Biv). Ca_V_2.2_HA was distributed around a central core of vGlut2 more frequently at DIV 28 compared to DIV 7 (Figure 1Bi and iv). Furthermore, the intensity of Ca_V_2.2_HA puncta, co-localized to vGluT2, normalized to the average intensity measured at DIV 7, was significantly higher at DIV 28 (5.9 ± 0.1) than at DIV 7 (1.0 ± 0.1) (Figure 1C). A gradual increase in Ca_V_2.2_HA intensity was also observed at the intermediate timepoints DIV 14 (2.3 ± 0.1) and DIV 21 (3.0 ± 0.1) (Figure 1Bii, Biii, and C).

### Development of Ca_V_2.2_HA Expression at Presynaptic Boutons of DRG—Spinal Cord Neuron Co-Cultures

**Figure 2.**
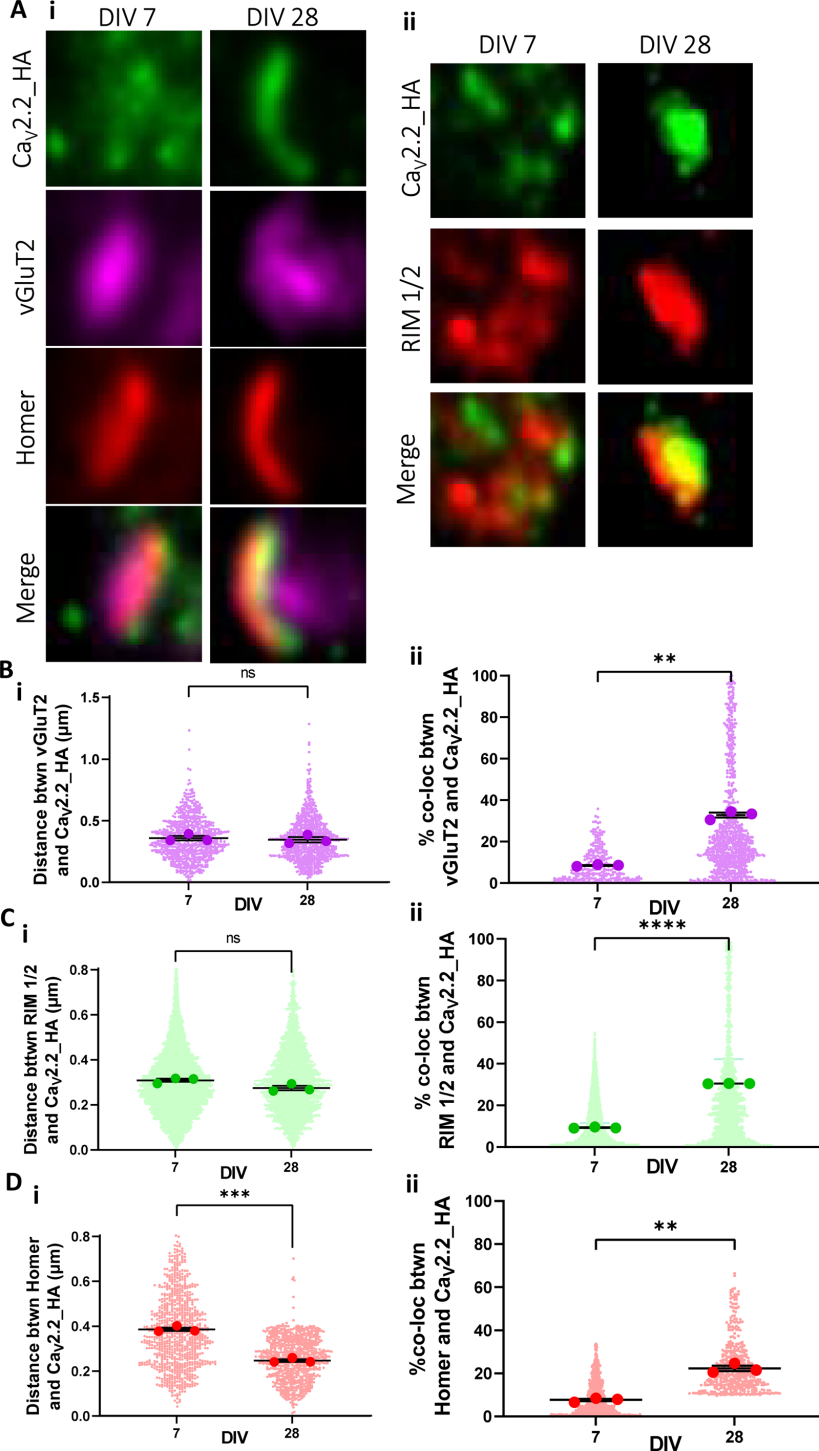
Increased co-localization of Ca_V_2.2_HA with vGluT2, Homer, and RM 1/2 at the presynaptic membrane in DRG-spinal cord co-cultures at DIV 28. (A) Representative images of single optical sections of synaptic puncta, (2 × 2 μm ROI), for DIV 7 (left) and DIV 28 (right) showing (i) Ca_V_2.2_HA (green), vGluT2 (magenta), Homer (red), and merged panels and (ii) Ca_V_2.2_HA (green), RIM 1/2 (red), and merged panels. Distance measurements between centres of co-localized objects (Bi) Ca_V_2.2_HA and vGluT2 (*P* = .672), (Ci) Ca_V_2.2_HA and RIM 1/2 (*P* = .052), and (Di) Ca_V_2.2_HA and Homer (*P* = .0002). Measurements of the percentage co-localizing volume for each object’s pair (Bii) Ca_V_2.2_HA and vGluT2 (*P* = .002), (Cii) Ca_V_2.2_HA and RIM 1/2 (*P* < .0001), and (Dii) Ca_V_2.2_HA and Homer (*P* = .003). Individual data points represent distance and percentage measurements between co-localizing objects, 900, 1400, and 1300 for Ca_V_2.2_HA and vGluT2, Ca_V_2.2_HA and RIM 1/2, and Ca_V_2.2_HA and Homer, respectively, from DIV 7 and 28. Mean of each experiment is shown with larger symbols. Mean ± SEM of the three experiments is superimposed. Statistical analysis: unpaired *t*-test with Welch’s correction; ****P < .0001, ***P < .001, **P < .01, ns = not significant.

Presynaptic Ca_V_2.2_HA was further examined using a panel of markers to study the temporal pattern of synapse formation between DRG and spinal cord neurons (Figure 2 and Supplementary Figure 1). We assessed the additional co-localization of Ca_V_2.2_HA with RIM 1/2 and Homer. Since Homer immunostaining reveals the majority of excitatory synapses,^36^ boutons were chosen based on the associated presence of Homer immunoreactivity. Furthermore, synaptic Ca_V_2.2 channels should be localized adjacent to docked and primed synaptic vesicles, and the presynaptic active zone was identified by the presence of RIM 1/2. At DIV 7, weak Ca_V_2.2_HA immunolabeling, associated with these synaptic markers, could be seen in immature cultures (Figure 2Ai and Aii). In contrast, in mature DIV 28 cultures, Ca_V_2.2_HA-positive puncta appeared clearly defined in close apposition to Homer, vGluT2, and RIM 1/2 puncta (Figure 2Ai and Aii). The appearance of intense punctate staining along the axonal processes was first seen at DIV 21 (Supplementary Figure 1). In addition to elongated punctate structures, Ca_V_2.2_HA can also be seen distributed around a central core of vGluT2 associated with Homer, resembling glomerular synapses.^37^ The rosette-shaped clusters of Ca_V_2.2_HA were comprised of up to five puncta (Supplementary Figure 1A and B). These patterns of immunoreactivity are consistent with our in-vivo study using Ca_V_2.2_HA^KI/KI^ mice.^25^ There was no difference between the relative distance between Ca_V_2.2_HA and vGluT2 puncta at DIV 7 (0.36 ± 0.02 μm and DIV 28 (0.35 ± 0.02 μm) (Figure 2Bi), or between Ca_V_2.2_HA and RIM 1/2 puncta at DIV 7 (0.31 ± 0.01 μm and DIV 28 (0.29 ± 0.01 μm (Figure 2Ci). However, a significant decrease was observed in the distance between Ca_V_2.2_HA and Homer at DIV 28 (0.24 ± 0.01 μm) compared to DIV 7 (0.38 ± 0.01 μm) (Figure 2Di), indicative of closer association with post-synaptic densities. Additionally, the overall percentage co-localization between Ca_V_2.2_HA and vGluT2, Homer, and RIM 1/2 increased by 22.6%, 14.0%, and 21.3%, respectively, when comparing DIV 7 to DIV 28 (Figure 2Bii, Cii, and Dii). This was also seen at the intermediate time point DIV 21 (Supplementary Figure 1C, D, and E). Taken together, these data suggest that the expression pattern of presynaptic Ca_V_2.2_HA in these co-cultures is developmentally regulated, consistent with the formation of mature synapses between DIV 21 and DIV 28.

### Development of Functional Synapses Dependent on N-type Channels in DRG—Spinal Cord Neuron Co-Cultures

**Figure 3.**
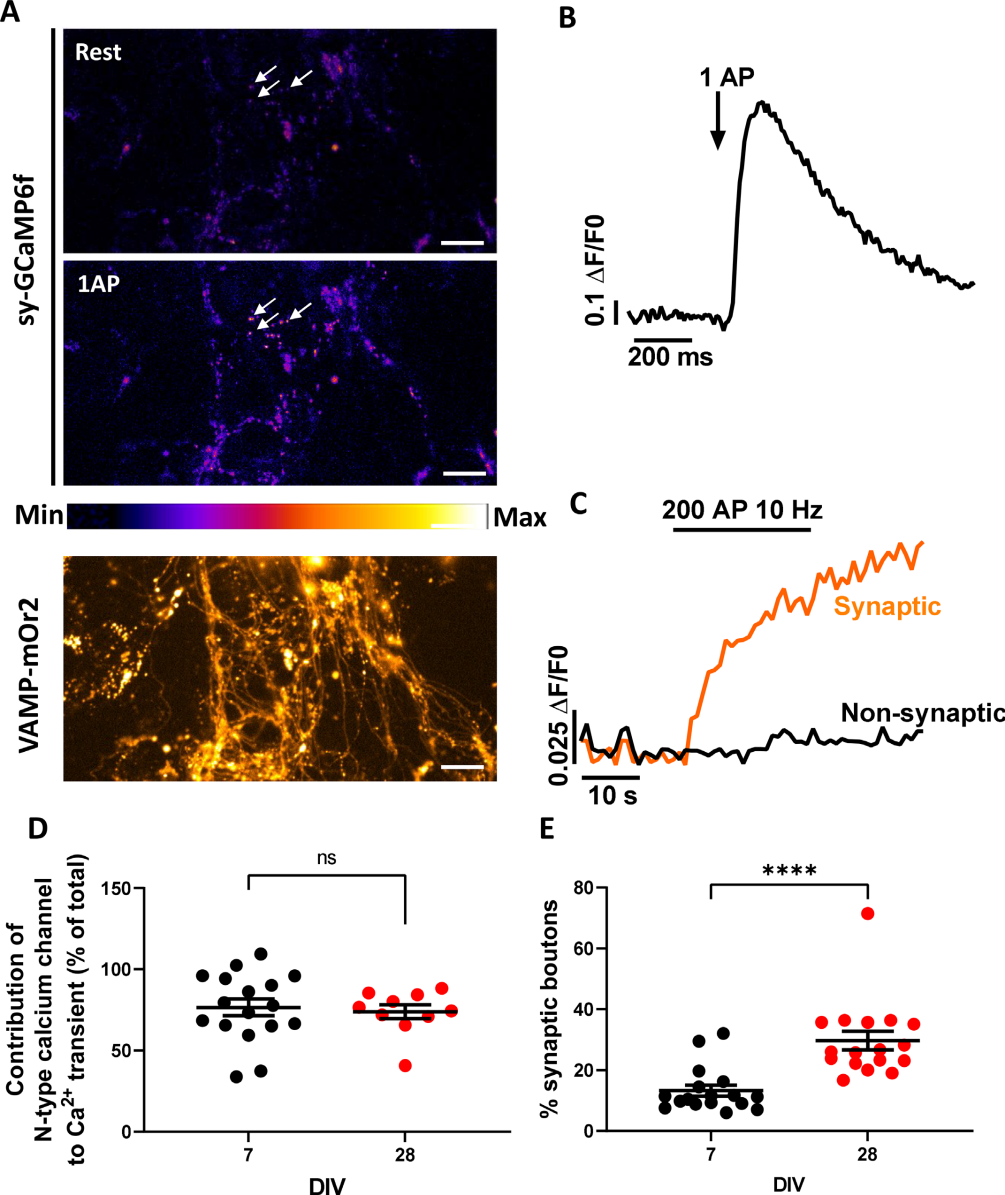
Increase in presynaptic Ca^2+^ transients associated with synaptic boutons relative to nonreleasing boutons in co-cultures between DIV 7 and 28. (A) GCaMP6f fluorescence changes in presynaptic terminals of DRG neurons expressing sy-GCaMP6f and VAMP-mOr2, in response to electrical field stimulation. Sy-GCaMP6f fluorescence: at rest (top) and after 1AP (middle) stimulation. White arrows point to examples of transfected boutons. The pseudocolor scale is shown below the second panel. The bottom panel shows presynaptic terminals expressing VAMP-mOr2 following 200 AP stimulation. Scale bar: 10 μm. (B) Example of sy-GCaMP6f fluorescence (Ca^2+^ transient) increase in response to 1AP stimulation in DRG neuron terminals (averaged trace from 5 neurons). (C) Examples of VAMP-mOr2 fluorescence in response to 200 APs at 10 Hz from DRG neuron terminals, used to identify vesicular release from presynaptic boutons: each individual bouton was analyzed and grouped into “nonsynaptic” (black trace) or “synaptic” (orange trace; positive response to 200 AP at 10 Hz) groups depending on whether there was no variation or an increase in fluorescence was recorded in response to stimulation. The traces correspond to the average responses from 50 boutons each. (D) Contribution of N-type VGCCs to the Ca^2+^ transient in response to 1 AP; 76.6% ± 5.1% (*n* = 17) and 73.9% ± 4.3% (*n* = 10) for DIV 7 (black circles) and 28 (red circles), respectively (measured from synaptic boutons). (E) % of synaptic-positive boutons at DIV 7 (black circles) and 28 (red circles), *n* = 17 for both DIV 7 and 28. *n* number refers to the number of experiments at each timepoint in vitro. Statistical analysis: Student’s *t*-test with Welch’s correction; ****P < .0001 and ns = not significant (*P* = .692).

After characterization of the developmental organization of endogenous Ca_V_2.2_HA at DRG presynaptic terminals within co-cultures, we next sought to establish whether these synaptic boutons were functional using Ca^2+^ imaging at increasing DIV. Dorsal root ganglion neurons were transfected, prior to plating, with the functional presynaptic reporter synaptophysin coupled to the genetically encoded Ca^2+^ indicator GCaMP6f (sy-GCaMP6f),^26^ and with a reporter of presynaptic exocytosis, vesicle-associated membrane protein (VAMP) tagged with the pH-sensitive fluorescent protein mOrange 2 (VAMP-mOr2) (Figure 3A). To assess local Ca^2+^ transients, in response to a single action potential (AP), a train of 1 AP stimuli was applied (Figure 3B). An increase of VAMP-mOr2 fluorescence in response to a stimulus of 200 APs at 10 Hz was then used to identify the responses from functional synaptic boutons, as previously described^29,38^ (Figure 3A and C). Additionally, to identify the contribution of N-type VGCCs to the Ca^2+^ transients, we used the specific Ca_V_2.2 inhibitor, ω-conotoxin GVIA (CTX, 1 µm). Application of CTX to co-cultures at DIV 7 and 28 indicated that N-type VGCCs mediated 76.6% ± 5.1% and 73.9% ± 4.3% of Ca^2+^ entry due to 1 AP, respectively (Figure 3D), with similar results at intermediate DIVs (Supplementary Figure 2A). However, there was a marked increase in the percentage of functional synaptic boutons at DIV 28 (30.2% ± 3.1%) compared to DIV 7 (13.4% ± 1.9%) (Figure 3E). A similar increase was observed at DIV 21 (Supplementary Figure 2B). These results suggest that DRG and spinal cord neurons readily form functional synapses with increasing time in co-culture, from DIV 7 to 28 and that N-type calcium channels are responsible for a large proportion of the Ca^2+^ transient seen following 1 AP stimulation.

### Cell Surface Ca_V_2.2_HA Is Preferentially Expressed in TRPV1-Positive DRG Neurons in Co-Cultures

Ca_V_2.2_HA distribution was also compared in DRG neuronal somata with respect to cell size and DIV (Supplementary Figure 3Ai and ii). A ring-like pattern of Ca_V_2.2_HA immunolabeling at the cell surface is observed in both small and medium DRG neurons, which diminished with increasing time in culture from DIV 7 to DIV 21–28 (Supplementary Figure 3A–C).

**Figure 4.**
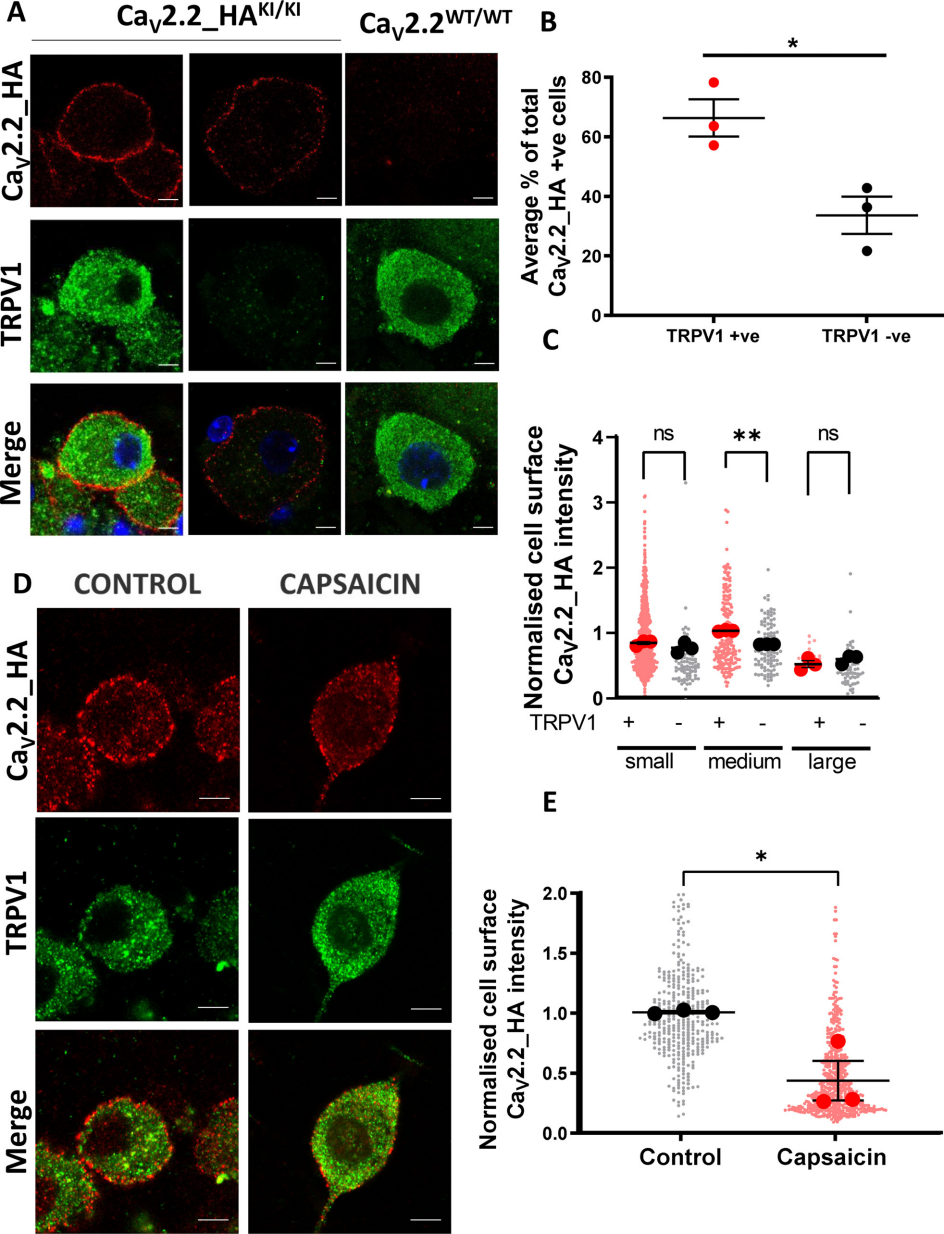
Decrease in cell surface Ca_V_2.2_HA in small DRG neurons following incubation with capsaicin. (A) Images of Ca_V_2.2_HA^KI/KI^ and Ca_V_2.2^WT/WT^ DRG neurons in DIV 21 co-cultures, showing Ca_V_2.2_HA staining before permeabilization (top; red), TRPV1 staining following permeabilization (middle; green), and merged images (bottom), for representative Ca_V_2.2_HA^KI/KI^ (left and middle panels), and Ca_V_2.2^WT/WT^ (right panel) DRG neurons. Scale bars 5 μm. (B) Quantification of the percentage of cells with cell-surface Ca_V_2.2_HA that were either positive (red circles) or negative (black circles) for TRPV1. Individual data points represent the mean data from three separate experiments and a total of 311 DRG neurons. *P < 0.021 (Student’s *t*-test). (***C***) Normalized cell surface Ca_V_2.2_HA intensity with respect to cell size: small, medium, and large DRG neurons that are either TRPV1-positive (red circles) or TRPV1-negative (black circles). Individual data points represent normalized Ca_V_2.2_HA intensity measured from all Ca_V_2.2_HA positive cells from three separate experiments (with mean of each shown in larger circles) and a total of 185, 77, 184, 102, 14, and 63 DRG neurons, respectively. Mean ± SEM of the three experiments is superimposed. Statistical analysis: one-way ANOVA with Sidak’s multiple selected comparison post-hoc test; **P = .0037, ns = not significant (*P* = .39, small and *P* = .37, large). (D) Capsaicin or control medium applied to DIV 21 co-cultures for 2 min, followed by 60 min rest at 37°C prior to fixation. Representative small DRG neurons in control (left) and capsaicin (right) conditions, showing Ca_V_2.2_HA (top; red), TRPV1 (middle; green), and merged image (bottom). Scale bars: 5 μm. (E) Normalized cell surface Ca_V_2.2_HA intensity measured from control or capsaicin-treated small DRG neurons following 60 min rest at 37°C. Individual data points represent normalized Ca_V_2.2_HA intensity measured from three separate experiments and a total of 357 and 546 small DRG neurons from control (black circles) and capsaicin conditions (red circles). Mean of each experiment shown in larger symbols. Mean ± SEM (*n* = 3) is superimposed. Statistical analysis: Student’s *t*-test *P = .0258.

Capsaicin sensitivity is an important pharmacological trait of a major subset of nociceptive sensory neurons, which mediates its effects through TRPV1.^13,39^ As described above, capsaicin has been reported to profoundly inhibit VGCC currents in DRG neurons.^40^ To understand the association between TRPV1 and Ca_V_2.2 in DRG populations, co-cultures were immunolabeled for both Ca_V_2.2_HA and TRPV1. There was a clear ring of Ca_V_2.2_HA at the plasma membrane of most neurons positive for TRPV1, and this was absent from Ca_V_2.2 wild-type neurons (Figure 4A). Although cell surface Ca_V_2.2_HA immunoreactivity can also be seen, to a lesser extent, on TRPV1-negative neurons (Figure 4A), 66.4% ± 6.3% of Ca_V_2.2_HA-positive neurons expressed TRPV1 (Figure 4B). TRPV1 expression was found to be highest in small and medium DRG neurons, as also seen in previous studies.^16,41^ Cell surface Ca_V_2.2_HA expression levels were higher, particularly in medium TRPV1-positive neurons, compared to their TRPV1-negative counterparts (Figure 4C). These data suggest that Ca_V_2.2_HA is preferentially expressed in TRPV1-positive medium DRG neurons.

### Capsaicin Reduces Somatic Cell Surface Expression of Ca_V_2.2_HA

To study the effect of capsaicin on cell surface Ca_V_2.2_HA expression and its time course, co-cultures were incubated with either 1 μm capsaicin or control buffer solution for 2 min at 37°C and then allowed to rest at 37°C for 0, 20, 40, or 60 min. Capsaicin (2 min) produced a dramatic reduction, by 57% in Ca_V_2.2_HA cell surface labeling in small DRG neurons after 60 min (Figure 4D and E) and after 40 min rest (Supplementary Figure 4). Moreover, a similar pattern was observed in medium DRG neurons (Supplementary Figure 5). In contrast, in controls, we observed no change in plasma membrane labeling of Ca_V_2.2_HA in either small or medium DRG neurons at any time point (Supplementary Figures 4A, C and 5A, C). These data indicate that capsaicin induces a decrease in cell surface Ca_V_2.2_HA expression in both small and medium DRG neurons, which occurs with a slow time-course.

### Capsaicin Modulates Presynaptic Ca_V_2.2_HA Expression in Co-Cultures

**Figure 5.**
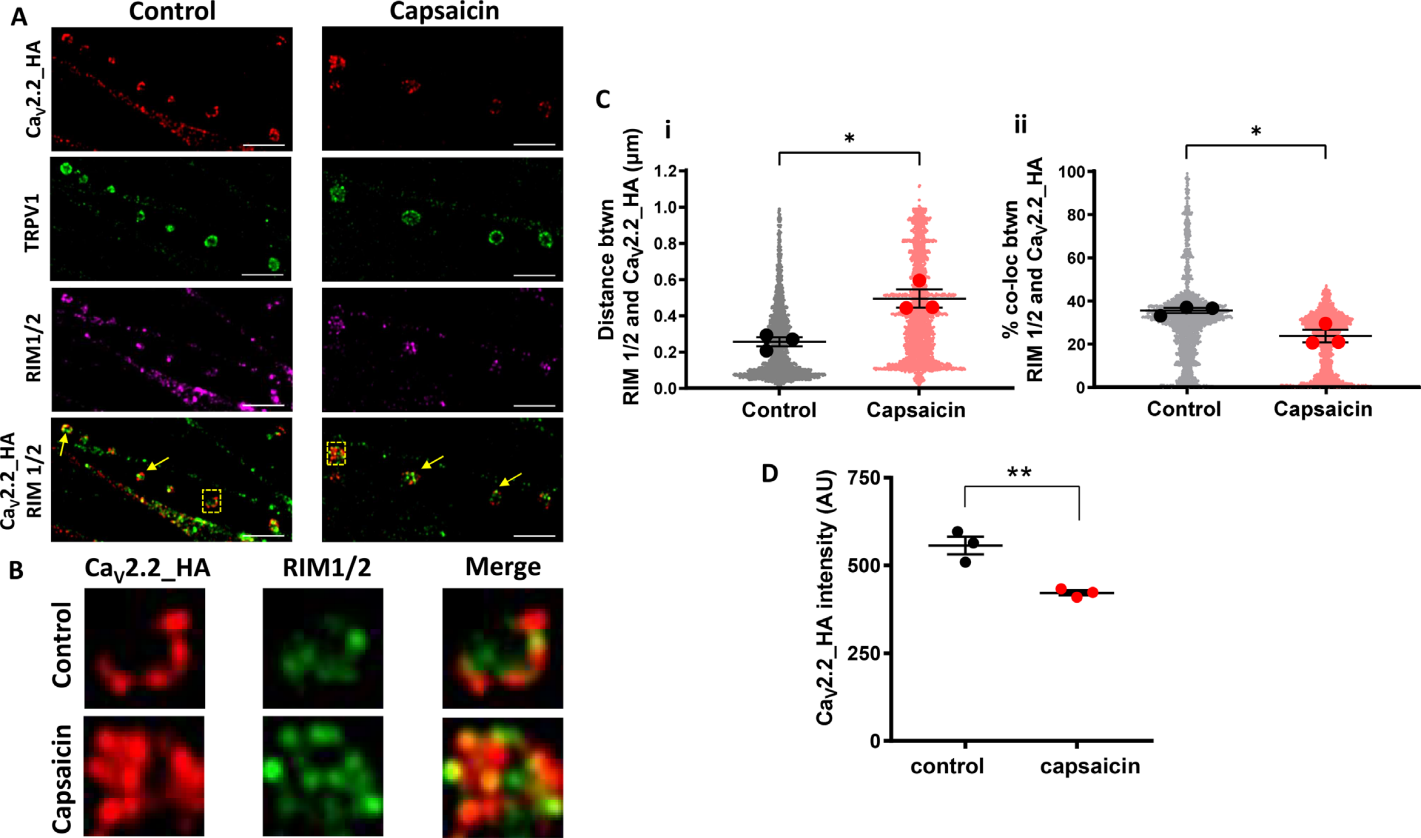
Decrease in Ca_V_2.2_HA immunolabeling in presynaptic terminals of DRG neurons following 2 min treatment with capsaicin. Capsaicin or control medium applied to DIV 21 co-cultures for 2 min, followed by 60 min rest at 37°C prior to fixation. (A) Images of presynaptic terminals from DRG neurons control (left) and capsaicin (right) conditions. Ca_V_2.2_HA (top row; red), TRPV1 (second row; green), RIM1/2 (third row; magenta), merged image of Ca_V_2.2_HA (red), and RIM 1/2 (recolored green for clarity; bottom row). Co-localized Ca_V_2.2_HA and RIM 1/2 marked by yellow arrows. Scale bars: 5 μm. (B) Images are enlargements of the ROI (2 × 2 μm dashed boxes) in (A), showing representative presynaptic terminals in control (top) and capsaicin (bottom) conditions. From left to right, Ca_V_2.2_HA (left; red), RIM 1/2 (middle; green) and merged panel (right). (Ci) Distance measurements between centres of co-localized objects Ca_V_2.2_HA and RIM 1/2 (*P = .013). (Cii) Measurements of the percentage co-localizing volume for each object’s pair for Ca_V_2.2_HA and RIM 1/2 (*P = .02). Individual data points represent distance and percentage measurements between co-localizing objects, 2300 pairs for Ca_V_2.2_HA and RIM 1/2, for both control and capsaicin conditions. Mean ± SEM of the three experiments are superimposed, with individual means as larger symbols. (D) Mean ± SEM of the three experiments showing intensity of Ca_V_2.2_HA puncta associated with RIM 1/2 (**P = .0068). Statistical analysis: Student’s *t*-test.

**Figure 6.**
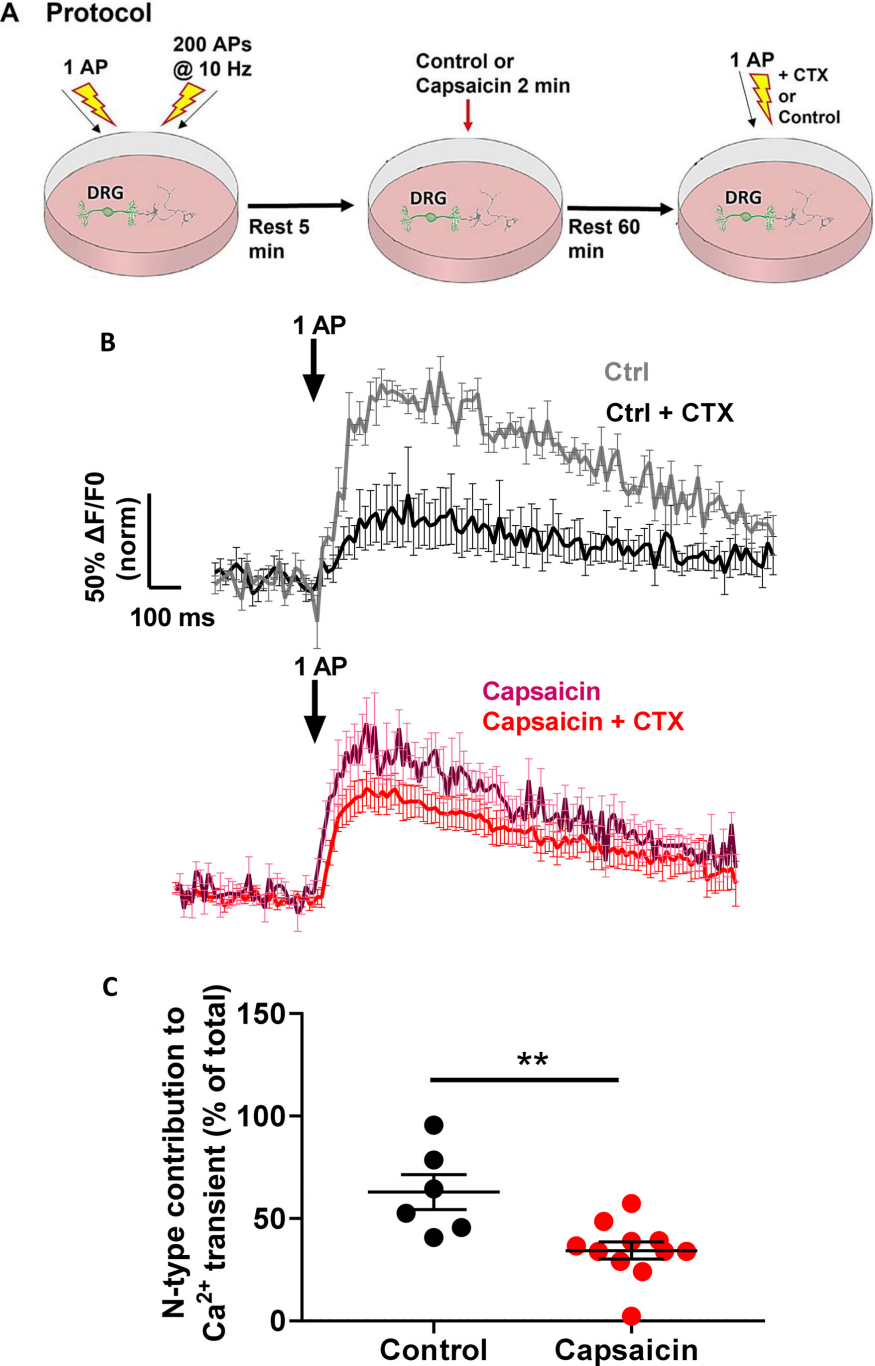
Reduction Ca_V_2.2 contribution to Ca^2+^ transients following 1 AP stimulation in capsaicin-treated, compared to control co-cultures following 60 min rest. (A) Schematic diagram of Ca^2+^ imaging protocol to test the effects of capsaicin in 21 DIV co-cultures. An initial protocol consisting of 1 AP and 200 APs (at 10 Hz) stimulations was applied to assess the baseline response of the neuron. Following a 5 min rest period, either control or capsaicin medium was applied for 2 min. Control medium was then applied for a 60 min rest period. CTX was then applied for 2 min. A final 1 AP stimulation was applied and the initial 1 AP Ca^2+^ transients were used to normalize the final 1 AP Ca^2+^ transients to determine the contribution of N-type calcium channels to the response. (B) Average normalized Sy-GCaMP6f fluorescence change in response to 1 AP stimulation, from control [top traces, pre-CTX gray (*n* = 8), post-CTX black (*n* = 8)] or capsaicin-treated [bottom traces, pre-CTX purple (*n* = 11), post-CTX red (*n* = 11)] neurons. The Ca^2+^ transients are expressed as ΔF/F0 and normalized to the averaged peak recorded from synaptic boutons before control medium/capsaicin and CTX was applied. Scale bars apply to both sets of traces. Mean values for peak Ca^2+^ transients are given in Supplementary Table 1. (C) Contribution of N-type voltage-gated calcium channels to the Ca^2+^ transient in response to 1 AP. For Control + CTX (black circles) *n* = 6, and for capsaicin + CTX (red circles) *n* = 11. Statistical analysis: Mann–Whitney test; **P = .0031.

We next examined the effect of capsaicin on expression of Ca_V_2.2_HA at DRG presynaptic terminals, using RIM 1/2 expression as a presynaptic marker. In DIV 21 co-cultures, Ca_V_2.2_HA and RIM 1/2 labeling can be seen along processes on control and capsaicin-treated neurons (Figure 5A). Enlargements from the Airyscan images (Figure 5A) of individual rosette clusters of Ca_V_2.2_HA puncta that co-localized with RIM 1/2 show clear differences between the capsaicin-treated and the control conditions, after 60 min of rest (Figure 5B). Analysis revealed that there was an increase in the relative distance between the centres of Ca_V_2.2_HA and RIM 1/2 in capsaicin-treated (0.49 ± 0.05 μm) compared to control (0.25 ± 0.03 μm) co-cultures (Figure 5Ci). Furthermore, at the same time point (60 min), there was a decrease in the overall percentage co-localization between Ca_V_2.2_HA and RIM 1/2 to 23.5% ± 2.9% in capsaicin-treated co-cultures, from 35.3% ± 1.2% in controls (Figure 5Cii). Although there was no significant increase in the relative distance between the centres of Ca_V_2.2_HA and RIM 1/2 at any other time point (Supplementary Figure 6A and B), a significant decrease in their percentage co-localization was also observed in capsaicin-treated neurons after 40 min (Supplementary Figure 6A and C). There was also a capsaicin-induced reduction in intensity of Ca_V_2.2_HA puncta associated with RIM 1/2 (Figure 6D).

### Capsaicin Disturbs the Contribution of the N-type Calcium Channel to Ca^2+^ Transients

To determine the effect of capsaicin on the function of presynaptic N-type calcium channels, AP-mediated presynaptic Ca^2+^ elevation was examined using sy-GCaMP6f. Co-cultures were first stimulated with a 1 AP train followed by 200 APs at 10 Hz to determine the baseline responses of the synaptic boutons. Following a 5 min rest period, co-cultures were perfused with 1 μm capsaicin or control medium for 2 min and then perfused for 20, 40, or 60 min with control medium. To determine the contribution of N-type calcium channels, co-cultures were then re-stimulated with 1 AP in the presence or absence of CTX (Figure 6A).

In control conditions, when neurons were perfused with control medium and allowed to rest for 60 min, we observed that CTX caused a large decrease of 63% in the peak Ca^2+^ transient (control: 100% ± 6.0%, *n* = 11 compared to control + CTX: 37.0% ± 3.0%, *n* = 6) (Figure 6B). However, when we compared the effects of CTX on capsaicin-treated co-cultures, it reduced the peak Ca^2+^ transient following a 1 AP stimulation by only 28% (capsaicin: 80.0% ± 4.0%, *n* = 8 compared to capsaicin + CTX: 57.0% ± 1.0%, *n* = 11) (Figure 6B). Thus, there was a significant reduction in the effect of CTX following incubation with capsaicin on the 1 AP induced Ca^2+^ transient (Figure 6C). A similar effect of capsaicin can be seen after 40 min but not after 20 min (Supplementary Figure 7). Together, these data suggest that capsaicin reduces, with a slow timescale, the available N-type calcium channels that functionally contribute to Ca^2+^ transients following 1 AP stimulation. As there is no consistent reduction of peak Ca^2+^ transient in response to 1 AP at 60 min after capsaicin (Supplementary Table 1), it is possible that some compensation occurs with respect to other calcium channels over time, but this has not been investigated here.

### Reduced Temperature Inhibits Capsaicin-Induced Loss of Cell Surface and Active Zone Ca_V_2.2_HA

**Figure 7.**
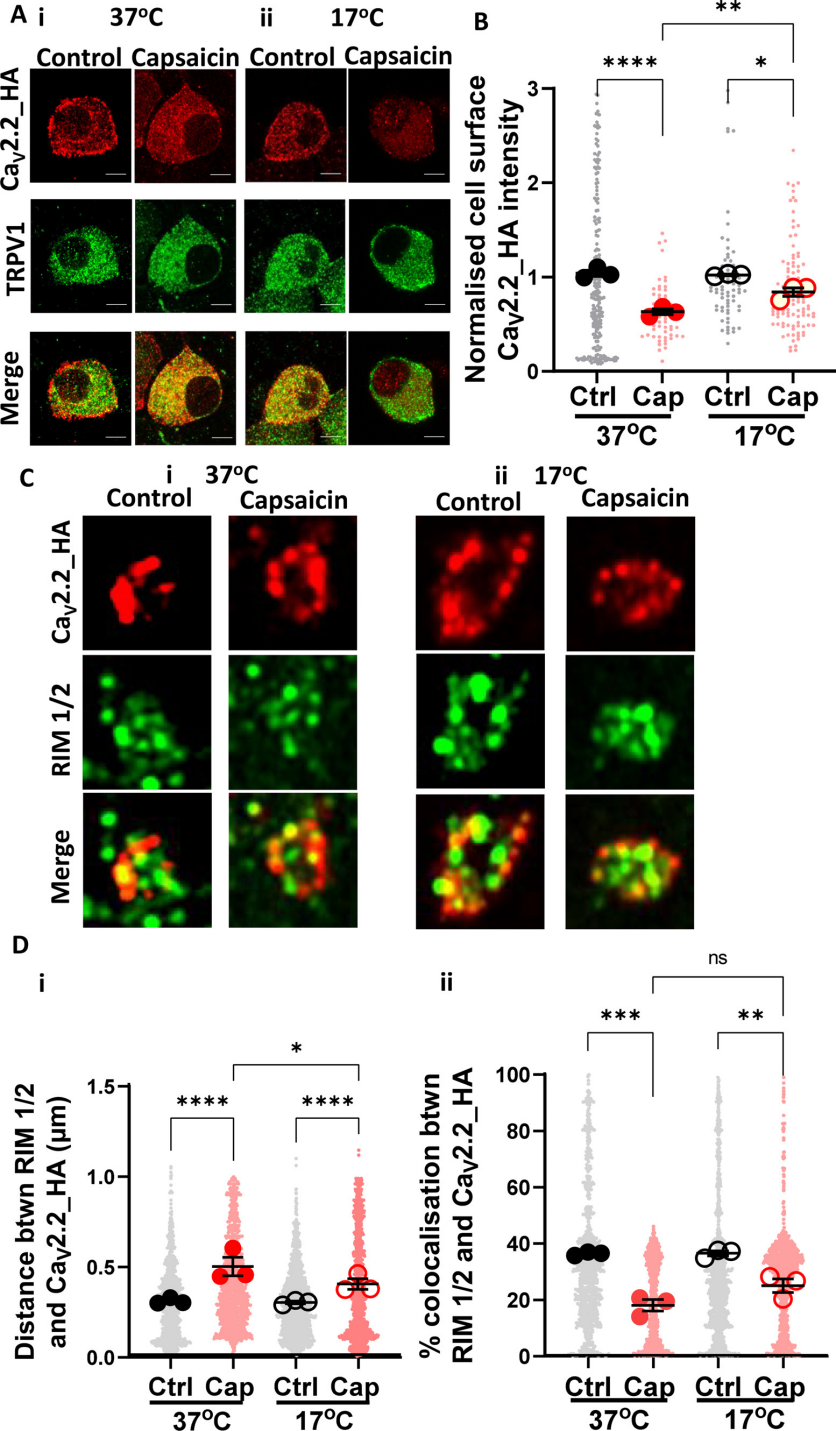
Decrease in cell surface Ca_V_2.2_HA in capsaicin-treated small DRG neurons and presynaptic terminals following incubation at 17°C. Capsaicin was applied to DIV 21 co-cultures for 2 min at 37°C, followed by 60 min rest at either 37°C or 17°C. (A) Images of small DRG neurons following (i) 37°C or (ii) 17°C rest period. Ca_V_2.2_HA (top; red), TRPV1 (middle; green), and merged panel (bottom). Scale bars: 5 μm. (B) Normalized cell surface Ca _V_2.2_HA intensity following 37°C (filled circles) or 17°C (open circles) rest. Individual data points represent Ca_V_2.2_HA intensity (normalized to mean in control conditions at 37°C), measured from three separate experiments and a total of 207 and 56 (control, gray) and 70, 106 (capsaicin, red) cells at 37°C and 17°C, respectively. Mean of each experiment shown by larger symbols. Mean ± SEM (*n* = 3) is superimposed. *P = .012, **P = .005, ****P < .0001. (C) Images (2 × 2 μm ROIs) of control (left) and capsaicin-treated (right) terminals following incubation for 60 min at either (i) 37°C or (ii) 17°C. Ca _V_2.2_HA (top; red), RIM 1/2 (middle; green), and merged panel (bottom). (Di) Distance between centres of co-localized objects Ca_V_2.2_HA and RIM 1/2 in control and capsaicin conditions following rest at either 37°C or 17°C. *P = .049, ****P < .0001. (Dii) Percentage co-localizing volume for each object’s pair for Ca_V_2.2_HA and RIM 1/2 in control and capsaicin conditions following rest at either 37°C or 17°C. **P < .0036, ***P < .0002, and ns = not significant. Data points for (D) represent distance and percentage measurements between individual co-localizing objects, 1500 pairs for Ca_V_2.2_HA and RIM 1/2, for control (black) and capsaicin (red) conditions at both temperatures. Mean of each experiment shown by larger symbols. Mean ± SEM (*n* = 3) superimposed. Statistical analysis for all graphs: one-way ANOVA with Sidak’s multiple comparison post-hoc test.

We then examined whether endocytosis of Ca_V_2.2_HA from the cell surface of DRG neurons was contributing to the effect of capsaicin, by employing a reduced temperature (17°C), which inhibits trafficking processes such as endocytosis. Capsaicin was applied to co-cultures for 2 min at 37°C, followed by rest for 60 min at either 37°C or 17°C. In small DRG neurons incubated at 37°C, a 37% decrease in cell surface Ca_V_2.2_HA immunolabeling was observed (control: 1.0 ± 0.03; capsaicin: 0.63 ± 0.03) (Figure 7A), similar to the results shown in Figure 4D. In contrast, when co-cultures were incubated at 17°C, a significantly smaller decrease of 16% in cell surface Ca_V_2.2_HA expression was measured (control: 1.0 ± 0.01; capsaicin: 0.84 ± 0.04) (Figure 7Aii and B). Similarly, in medium DRG neurons, capsaicin treatment resulted in a smaller decrease in cell surface Ca_V_2.2_HA expression at 17°C compared to 37°C (Supplementary Figure 8).

We next investigated the effect of capsaicin on expression of Ca_V_2.2_HA at presynaptic terminals following rest for 60 min at either 37°C or 17°C. At 37°C, there was a decrease in co-localization between Ca_V_2.2_HA and RIM 1/2 (Figure 7C and D), as previously observed. The distance between Ca_V_2.2_HA and RIM 1/2 was significantly increased by 63% from 0.30 ± 0.01 μm to 0.49 ± 0.05 μm (Figure 7Di). In contrast, at 17°C the distance between the two centres increased to a significantly smaller extent, by 34% (0.29 ± 0.01 μm to 0.39 ± 0.03 μm) (Figure 7C and Di). However, there was a nonsignificant reduction in percentage co-localization of Ca_V_2.2_HA and RIM 1/2 due to capsaicin at 37°C (a 50.8% decrease) and at 17°C (a 31.7% decrease) (Figure 7Dii). These data suggest that incubation at 17°C may impact Ca_V_2.2_HA distribution at both the cell body and presynaptic terminals by affecting a temperature-dependent process, such as endocytosis, induced by capsaicin.

### Dominant-Negative Rab11a Inhibits Capsaicin-Induced Loss of Functional Ca_V_2.2_HA in DRG Terminals

**Figure 8.**
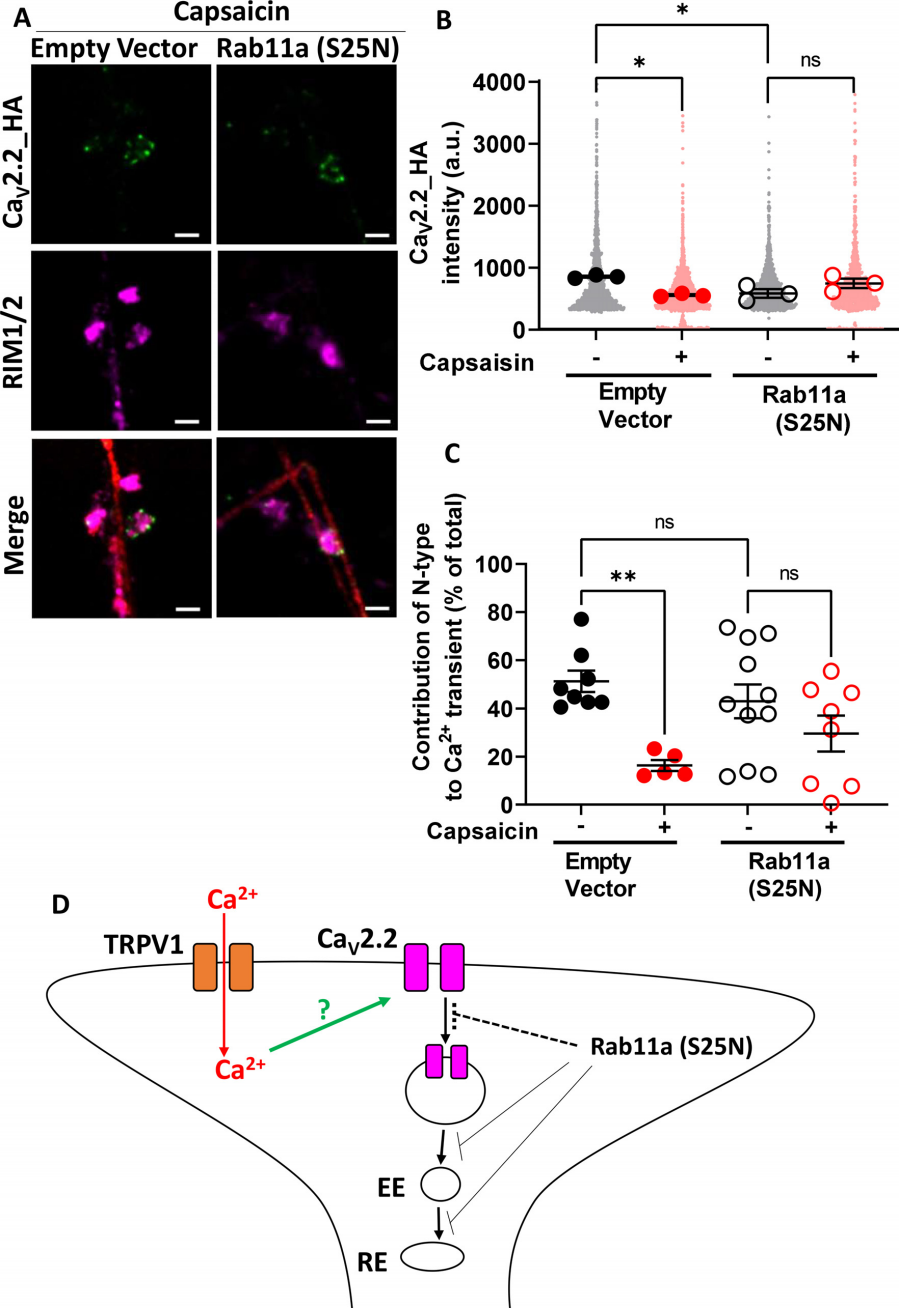
Effects of capsaicin on Ca_V_2.2_HA expression and presynaptic function are blocked by expression of dominant-negative Rab11a in co-cultured DRG neuron terminals. (***A***) Representative images showing capsaicin-treated co-cultures transfected with either empty vector (left) or Rab11a (S25N) (right). Ca_V_2.2_HA (green; top), RIM 1/2 (magenta; middle), and merged panel (bottom, including mCherry transfection marker). Scale bars 2 μm. (B) Mean Ca_V_2.2_HA intensity measured in presynaptic terminals (identified by presence of RIM 1/2) of DRG neurons transfected with either empty vector (left, solid symbols) or Rab11a (S25N) (right, open symbols) and treated with either control medium (black circles) or capsaicin (red circles). Individual data points represent 1400 puncta from all conditions, with means of each experiment in larger symbols. Mean ± SEM (*n* = 3) is superimposed. Statistical analysis: one-way ANOVA with Sidak’s multiple comparisons post-hoc test; *P = .0127 (EV +/- capsaicin), *P = .0213 [EV vs Rab11a (S25N)], and ns = not significant (*P* = .182). (C) Average normalized Sy-GCaMP6f fluorescence change in response to 1 AP stimulation recorded from synaptic boutons (DIV 21). Co-cultures were treated with either control medium (black circles) or capsaicin (red circles) followed by 60 min rest in control medium. Subsequently, CTX was applied, as in Figure 6A. The Ca^2+^ transient is expressed as ΔF/F0 and normalized to the averaged initial peak in response prior to capsaicin. For Empty Vector transfected cells, control + CTX (*n* = 8; filled black circles) and capsaicin + CTX (*n* = 5; filled red circles), and for Rab11a (S25N) transfected cells, control + CTX (*n* = 11; open black circles) and Capsaicin + CTX (*n* = 8; open red circles). Statistical analysis: one-way ANOVA with Sidak’s multiple comparison post-hoc test; **P = .008 and ns = not significant. Mean values for peak Ca^2+^ transients are given in Supplementary Table 1. (D) Schematic diagram for Ca_V_2.2 membrane expression and recycling via Rab11a-positive endosomes. EE = early endosome, RE = recycling endosome. Dotted pathways represent hypothetical routes.

We have previously shown that α_2_δ-1 and α_2_δ-2 are recycled to the plasma membrane via a Rab11a-dependent recycling endosome pathway.^11,12^ Furthermore, α_2_δ-1 increases Ca_V_2.2 at the plasma membrane by increasing the rate of the net forward trafficking of Ca_V_2.2 in a Rab11a-dependent manner.^12^ To test the involvement of Rab11a on the distribution of Ca_V_2.2_HA and the effect of capsaicin in co-cultures, we transfected DRG neurons with dominant-negative Rab11a (S25N), as previously described for hippocampal neurons.^11,12^ We compared the effects of capsaicin on presynaptic Ca_V_2.2_HA expression in the absence and presence of Rab11a (S25N), using mCherry as a transfection marker (Figure 8A). Similar Ca_V_2.2_HA immunolabeling was observed, apposed to RIM 1/2, at the presynaptic active zone when capsaicin was applied to co-cultures expressing either Rab11a (S25N) or empty vector (Figure 8A). In control-transfected neurons expressing mCherry, we observed a 34% decrease in Ca_V_2.2_HA immunostaining puncta associated with RIM 1/2 as a result of capsaicin treatment in the DRG terminals (Figure 8B). In contrast, in DRG terminals expressing Rab11a (S25N), there was no significant difference in Ca_V_2.2_HA expression in the presence or absence of capsaicin (Figure 8B). These data, supported by those in Figure 7, provide evidence that presynaptic Ca_V_2.2_HA distribution is regulated by a Rab11a-dependent process, potentially involving capsaicin-induced endocytosis.

This result was supported by the measurement of AP-mediated presynaptic Ca^2+^ transients. The protocol outlined in Figure 6A was applied to co-cultures of DRG neurons transfected with Rab11a (S25N) or control empty vector. Similar to the results seen in Figure 6B and C, following the application of capsaicin, the N-type channel contribution to the 1 AP Ca^2+^ transient was markedly reduced from 51.3% ± 4.4% (control) to 16.4% ± 2.3% (capsaicin) for control-transfected DRGs (Figure 8C). In contrast, in co-cultures expressing Rab11a (S25N), capsaicin produced no significant difference in the contribution of the N-type calcium channels to 1 AP-induced Ca^2+^ transients (control: 40.9% ± 8.2%, compared to capsaicin: 29.4% ± 7.6%). Together, these results suggest that interference with Rab11a-dependent function at presynaptic terminals may reduce endocytosis of Ca_V_2.2_HA in response to capsaicin.

## Discussion

### Ca_V_2.2_HA Expression at the Presynaptic Boutons of DRG—Spinal Cord Neuron Co-Cultures

Co-cultures of dissociated DRG and spinal cord neurons have been used as a model system to characterize the properties of primary afferent synapses.^21,22^ Importantly, DRG neurons do not form synapses between each other in vivo or in culture. In the present study, co-cultures were established by using DRG neurons from P0/P1 Ca_V_2.2_HA^KI/KI^ mice, cultured with spinal cord neurons from Ca_V_2.2^WT/WT^ mice. Thus, DRG neurons and their terminals could be distinguished based on the presence of Ca_V_2.2_HA and cell body size.

Our work first details the development of endogenous Ca_V_2.2_HA distribution during synapse formation between DIV 7 and 28, in these co-cultures. Dorsal root ganglion neuron terminals were detected using Ca_V_2.2_HA, together with presynaptic markers vGluT2 and RIM 1/2, together with the postsynaptic marker, Homer (Figures 1 and 2). The predominant presynaptic boutons on central processes of primary afferents are of the *en passant* type, and have a diameter of 1–2 μm and length of 1–4 μm.^42,43^ In the co-culture system described, axonal boutons of similar sizes were observed.

We find a significant decrease in cell surface expression of Ca_V_2.2_HA at the cell body of small and medium DRG neurons in co-cultures over time (Supplementary Figure 3). In parallel, a significant increase of Ca_V_2.2_HA expression was observed at DRG presynaptic terminals within the co-cultures (Figure 1). After protein synthesis in neuronal somata, several mechanisms have been proposed to mediate the delivery of membrane proteins to axons. These include axonal transport within trafficking endosomes, and non-polarised delivery to the somatic membrane, followed by transcytosis and endosomal transport in axons.^44,45^ For example, it has been shown that trkA receptors are transported to axons from the soma by transcytosis during development of sympathetic neurons.^46^ The decrease in Ca_V_2.2_HA at the somatic plasma membrane (Supplementary Figure 3) may be due to redirection of channels into axons of the developing neurons. Another method of presynaptic protein delivery is through local synthesis of proteins at axonal sites.^47^ However, further studies are required to determine whether any Ca_V_2.2 is synthesized locally in DRG terminals.

### Synaptic Boutons of DRG—Spinal Cord Neuron Co-Cultures form Functional Synapses

Native VGCCs have been classified in DRG neurons using specific pharmacological agents to elucidate their physiological contribution.^48–50^ Dorsal root ganglion neurons have been shown to express N-type channels, as well as other calcium channels in differing proportions.^2,51^ In the present study, Ca^2+^ transients were recorded from co-cultures following a train of 1 AP stimuli (Figure 3). In parallel with the changes in synaptic morphology, there was a corresponding increase in the number of functional synaptic boutons between DIV 7 and 28 (Figure 3). To further dissect the contribution of Ca_V_2.2 channels to the Ca^2+^ transients, CTX was found to significantly reduce the amplitude of the 1 AP-induced Ca^2+^ transient, which is consistent with the previously described important role of N-type VGCCs in triggering glutamate release at primary afferent synapses.^52,53^ However, further studies are required to determine the contribution of other VGCCs to these Ca^2+^ transients.

### Capsaicin Modulates Presynaptic Ca_V_2.2_HA Expression in Co-Cultures

The effects of capsaicin on VGCC expression in different cell types, and the mechanisms involved, remain unclear. Capsaicin has been found to indirectly reduce Ca^2+^ entry through VGCCs in rat trigeminal and hippocampal neurons,^54^ and gastric smooth muscle.^55^ Similarly, in rat sensory neurons, TRPV1 activation, through capsaicin, mediates an overall reduction in N-type calcium current.^20,56^ In contrast, in guinea pig DRG neurons, capsaicin shifts the VGCC current-voltage relationship to more hyperpolarized potentials, which would result in facilitated activation of VGCCs in response to smaller depolarization.^57^

To understand the effect of capsaicin on Ca_V_2.2 channels, and its consequences for primary afferent transmission, we examined the impact of capsaicin on cell surface Ca_V_2.2_HA expression. We found firstly that cell surface Ca_V_2.2_HA was predominantly present on small and medium TRPV1-positive DRG neurons (Figure 4). Secondly, we found a significant reduction in cell surface Ca_V_2.2_HA in these TRPV1-positive neurons in response to brief incubation with capsaicin, which is detected 40–60 min later (Figure 4 and Supplementary Figures 4 and 5). Previous studies have used a polyclonal antibody raised against an intracellular epitope in the II–III loop of Ca_V_2.2 to monitor potential internalization of the N-type calcium channel in rat DRG neurons in response to capsaicin.^20^ However, as this antibody cannot distinguish cell surface and intracellular Ca_V_2.2 expression, it is difficult to conclusively determine internalization of Ca_V_2.2 using such immunocytochemical methods.

Furthermore, in response to capsaicin we found a concomitant decrease in co-localization of Ca_V_2.2_HA with RIM 1/2 associated with presynaptic active zones (Figure 5). In parallel, the N-type calcium channel contribution to 1 AP Ca^2+^ transients was decreased following capsaicin application (Figure 6). The entry of Ca^2+^ through TRPV1 channels initiates a cascade of events within neurons. Wu et al. (2005) observed capsaicin-induced dephosphorylation of Ca_V_2.2 through Ca^2+^-dependent activation of calcineurin in rat DRG neurons. The role of calcineurin in Ca^2+^-dependent regulation of Ca^2+^ influx has previously been examined in NG108-15 cells.^58^ These authors demonstrated a decrease in high-voltage activated (HVA) current when calcineurin was over-expressed, which was reversed by intracellular FK506 (calcineurin inhibitor), attesting to calcineurin-dependency. Furthermore, the effect was blocked by the Ca^2+^ chelator BAPTA, highlighting that it is a Ca^2+^-dependent mechanism.^58^

### Dominant-Negative Rab11a Inhibits Capsaicin-Induced Endocytosis of Ca_V_2.2_HA in DRG Neurites

We next explored the involvement of endocytosis in the mechanism of action of capsaicin on presynaptic Ca_V_2.2_HA, by employing two strategies, reduced temperature (Figure 7) and dominant-negative Rab11a (S25N) (Figure 8). Rab11a belongs to the Rab family of small GTPases which are involved in many aspects of vesicular transport, via temporal and spatial interactions with multiple effectors,^59^ and Rab11 is required for the direct recycling of endosomes.^60^ It has been identified in many neuronal types as mainly residing in somatodendritic compartments,^61,62^ but it is also present in synaptic vesicles.^63^ The abrogation of Rab11a function by a dominant-negative form Rab11a (S25N)^64^ has been shown to disrupt the ability of α_2_δ-1 to increase Ca_V_2.2 expression in hippocampal neurites.^12^ Furthermore, Rab11 facilitates activity-dependent bulk endocytosis,^65^ which suggests it has a vital role in neurotransmission during intense neuronal activity and Ca^2+^ influx. Rab11-dependent recycling of Ca_V_2.2 may therefore contribute to the dynamic control of expression of Ca_V_2.2-HA in presynaptic terminals of DRG neurons following TRPV1 activation.

Our data indicate that blockade of Rab11-dependent processes with Rab11a (S25N) leads to a reduction in cell surface Ca_V_2.2_HA levels that are associated with RIM 1/2 in presynaptic terminals, and capsaicin is no longer able to exhibit its down-regulatory effects on presynaptic Ca_V_2.2_HA (Figure 8A and B). Furthermore, Rab11a (S25N) also prevented the capsaicin-induced reduction of the contribution of N-type calcium channel to 1 AP Ca^2+^ transients (Figure 8C and D). Our results using a lowered incubation temperature of 17°C also point to the involvement of Ca_V_2.2_HA endocytosis in response to capsaicin (Figure 7).

Previous work from our laboratory has shown that α_2_δ-1 and α_2_δ-2, but not α_2_δ-3 are recycled through Rab11a-dependent recycling endosomes and this process can be interrupted by gabapentin.^11,12^ Furthermore, in primary hippocampal neurites, Ca_V_2.2 membrane expression was found to be strongly dependent on the presence of an α_2_δ, and blockade of Rab11a-dependent recycling reduced cell surface Ca_V_2.2 levels in the presence of α_2_δ-1.^12^ Our observations here extend these findings and show that expression of Rab11a (S25N) reduces levels of native Ca_V_2.2_HA in DRG neuronal presynaptic terminals (Figure 8B). It is also worth noting that Rab11-dependent recycling is an important mechanism by which cell surface expression of other ion channels is modulated, including K_V_1.5, KCNQ1, and epithelial TRPV5 channels.^66–68^

The ability to examine plasma membrane expression and function of Ca_V_2.2 at presynaptic sites in the primary afferent pathway is critical for furthering our understanding of chronic pain and may suggest future routes for therapeutic targeting of this channel. Our results show that the use of DRGs from Ca_V_2.2_HA^KI/KI^ mice in co-culture with spinal cord neurons can be used to successfully examine the dynamic function and distribution of presynaptic Ca_V_2.2 channels and reveal the effect of TRPV1 activation on cell surface and presynaptic Ca_V_2.2_HA expression. Additionally, these data indicate that one of the main drivers of capsaicin-mediated decreases in Ca_V_2.2_HA from the plasma membrane involves a Rab11a-dependent process.

## Supplementary Material

zqac058_Supplemental_File

## Data Availability

The data underlying this article will be shared on reasonable request to the corresponding author.
