# Supplementary material for: Capsaicin-Induced Endocytosis of Endogenous Presynaptic CaV2.2 in DRG-Spinal Cord Co-Cultures Inhibits Presynaptic Function
Source: Function (Oxf). 2022 Nov 25;4(1):zqac058. doi: 10.1093/function/zqac058 (PMC9761886; doi:10.1093/function/zqac058)
Supplement: zqac058_Supplemental_File [file zqac058_supplemental_file.doc]

**Capsaicin-induced endocytosis of endogenous presynaptic Ca_V_2.2 in DRG-spinal cord co-cultures inhibits presynaptic function**

Krishma H Ramgoolam^1^ and Annette C Dolphin* ^1^

**Supplementary Table 1**

**Peak Ca^2+^ transients in response to 1 AP for the data shown in Figures (60 min after capsaicin)**

**Figure 6B**

| **Condition** | **ΔF/F0** | **SEM** | **N** |
| --- | --- | --- | --- |
| **Control** | 1.05 | 0.10 | 8 |
| **Control + Ctx** | 0.37 | 0.12 | 8 |
| **Capsaicin** | 0.71 | 0.05 | 11 |
| **Capsaicin + Ctx** | 0.63 | 0.04 | 11 |

**Figure 8**

| **Condition (EV)** | **ΔF/F0** | **SEM** | **N** |
| --- | --- | --- | --- |
| **Control** | 0.99 | 0.07 | 6 |
| **Control + Ctx** | 0.49 | 0.04 | 8 |
| **Capsaicin** | 0.94 | 0.07 | 10 |
| **Capsaicin + Ctx** | 0.83 | 0.02 | 5 |

| **Condition (Rab11aS25N)** | **ΔF/F0** | **SEM** | **N** |
| --- | --- | --- | --- |
| **Control** | 0.96 | 0.01 | 8 |
| **Control + Ctx** | 0.60 | 0.09 | 11 |
| **Capsaicin** | 0.94 | 0.12 | 8 |
| **Capsaicin + Ctx** | 0.21 | 0.08 | 8 |

**Supplementary Figures**

**
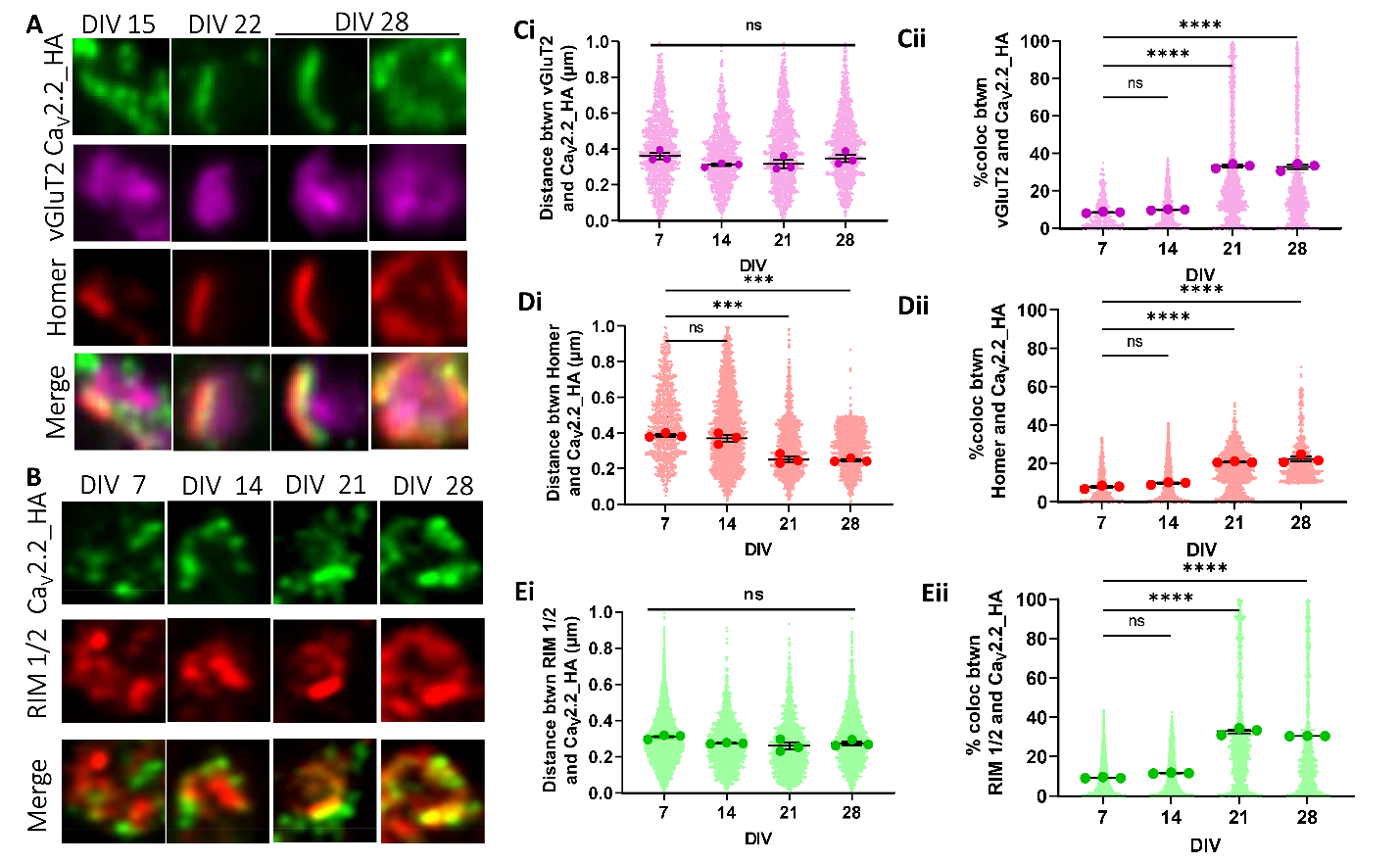
**

**Supplementary Figure 1: Increased co-localization of Ca_V_2.2_HA with vGluT2 and Homer at the presynaptic membrane in DRG - spinal cord co-cultures at DIV 21-28**

(**A, B**) Representative images (2 x 2 µm) of synaptic puncta, (**A**) shows (top to bottom) Ca_V_2.2_HA (green), vGluT2 (magenta), Homer (red) and merged panels, fixed (left to right) at DIV 15, 22 and 28 (two images). (**B**) shows (top to bottom) Ca_V_2.2_HA (green), RIM 1/2 (red) and merged panels, fixed (left to right) at DIV 7, 14, 21 and 28. (**Ci -Ei**) Distance measurements between centres of co-localized objects for (**Ci**) Ca_V_2.2_HA and vGluT2, (**Di**) Ca_V_2.2_HA and Homer and (**Ei**) Ca_V_2.2_HA and RIM 1/2. (**Cii -Eii**) Measurements of the percentage co-localizing volume for each object’s pair for (**Cii**) Ca_V_2.2_HA and vGluT2, (**Dii**) Ca_V_2.2_HA and Home and (**Eii**) Ca_V_2.2_HA and RIM 1/2. Individual data points represent distance and percentage measurements between co-localizing objects, 1200, 2300 and 1400 for Ca_V_2.2_HA and vGluT2 and Ca_V_2.2_HA and Homer, respectively from DIV 7, 14-15, 21-22 and 28 for 3 separate experiments. Mean of each experiment is shown in larger symbols. Mean ± SEM (n = 3) is superimposed. Statistical analysis: one-way ANOVA with Tukey selected comparisons post hoc test; ****p<0.0001, ***p<0.001, ns = not significant.

**
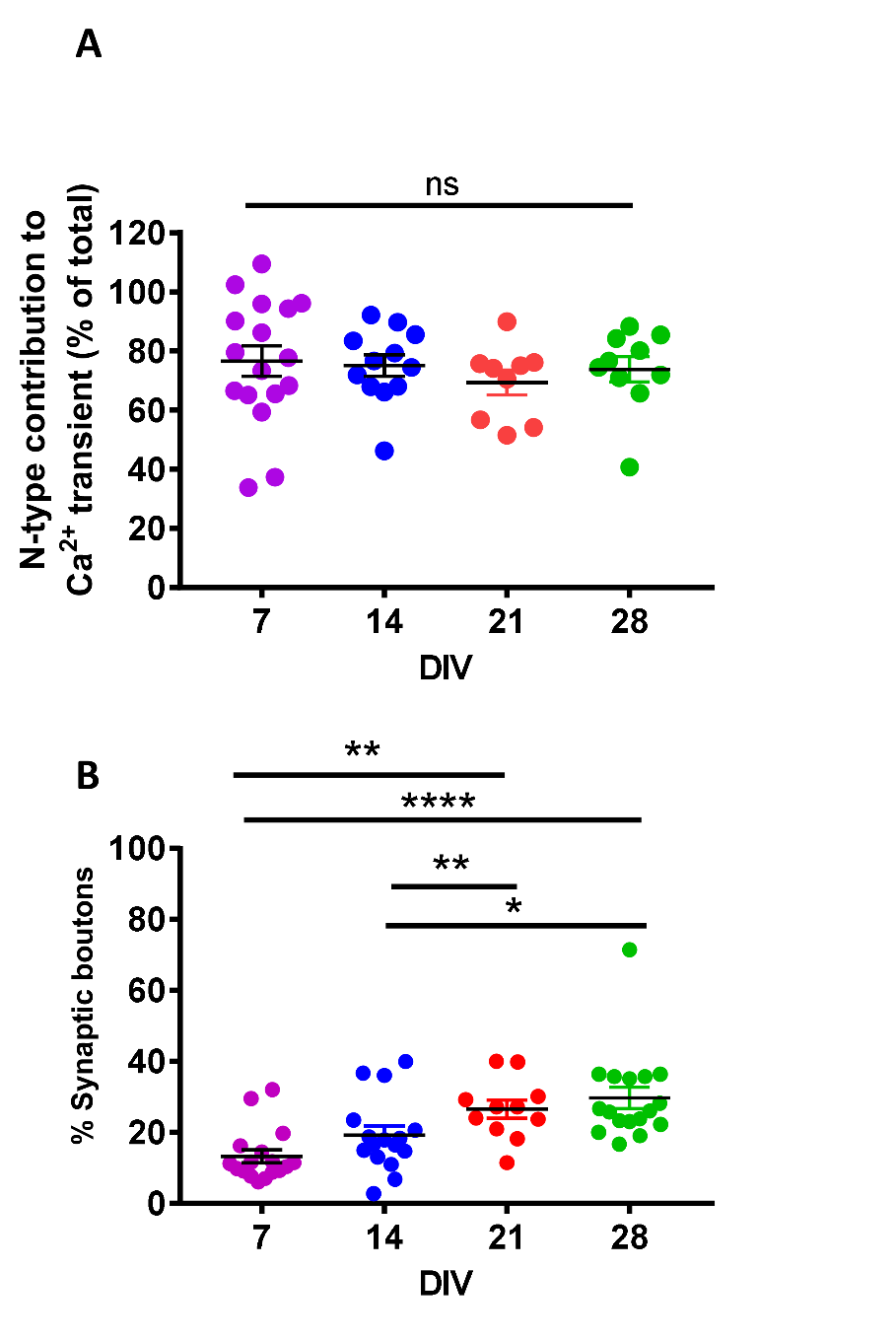
**

**Supplementary Figure 2: Properties of presynaptic 1 AP Ca^2+^ transients in DRG neuron terminals from co-cultures using Sy-GCaMP6f and VAMP-mOr2.**

(**A**) CTX was used to determine the N-type VGCC contribution to Ca^2+^ transients in response to 1 AP stimulation of co-cultures at increasing DIV. N-type VGCC contribution was 76.6 ± 5.1 % (n = 17), 75.1 ± 3.6 % (n = 12), 69.3 ± 4.2 % (n = 9) and 73.9 ± 4.3 % (n = 10), for DIV 7 (magenta), 14 (blue), 21 (red) and 28 (green), respectively. **(B**) % of VAMP-positive boutons at DIV 7 (n = 17; magenta), 14 (n = 13; blue), 21 (n = 11; red) and 28 (n = 17; green). Statistical analysis for (**A**) and (**B**): one-way ANOVA with Tukey post hoc test. For (A): ns, not significant for all comparisons. For (B): DIV 7 vs DIV 14, ns p = 0.331; DIV 7 vs DIV 21, ** p = 0.0064; DIV 7 vs DIV 28, **** p < 0.0001; DIV14 vs DIV 21, p = 0.260; DIV 14 vs DIV 28, * p = 0.0204; DIV 21 vs DIV 28, ns p = 0.844.


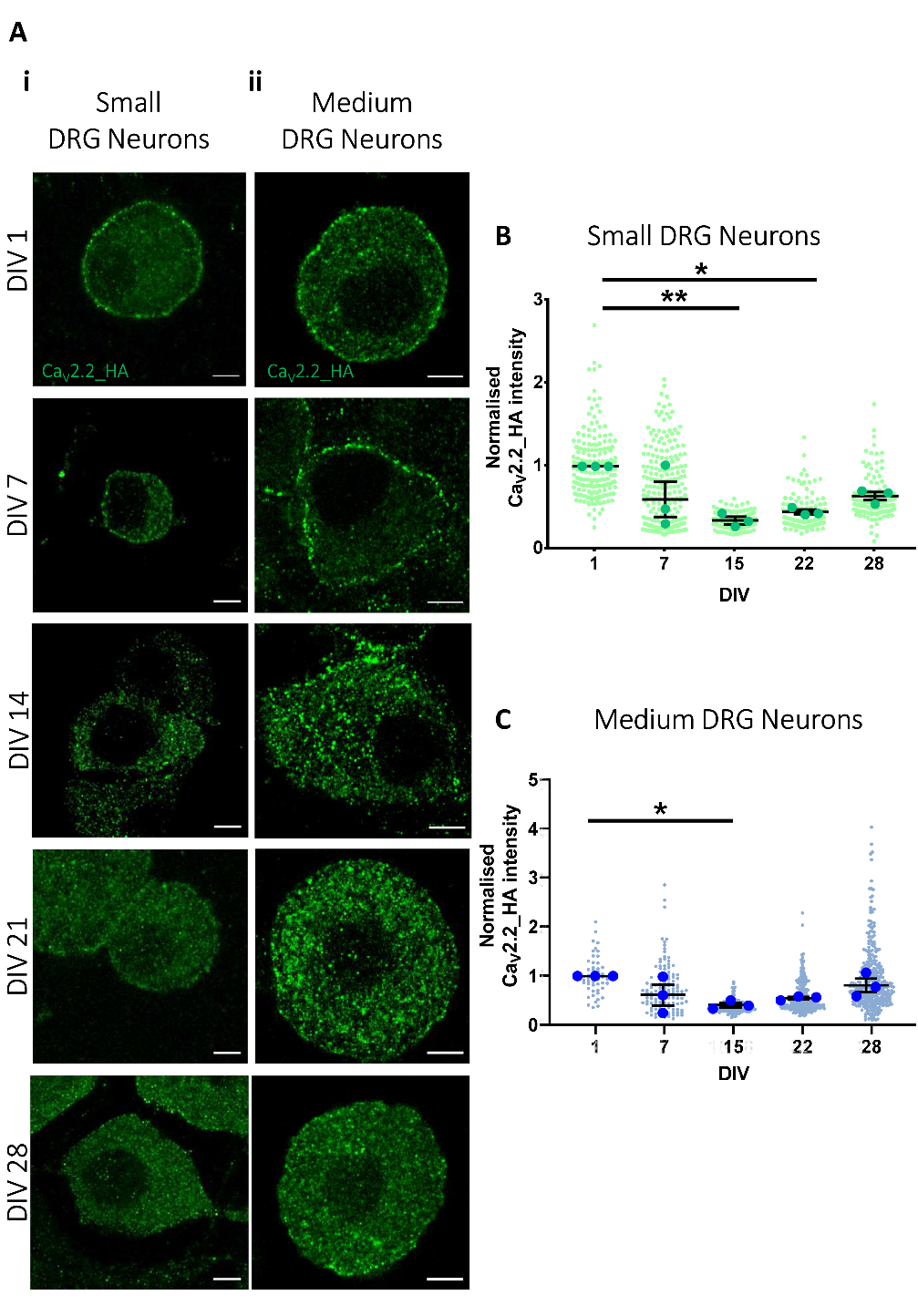


**Supplementary Figure 3: Cell surface Ca_V_2.2_HA expression on DRG cell bodies in co-cultures decreases over time *in vitro*.**

(**Ai**) Small and (**Aii**) medium DRG neurons from co-cultures composed of DRG neurons from Ca_V_2.2_HA^KI/KI^ and spinal cord neurons from Ca_V_2.2^WT/WT^ mice. Airyscan images of Ca_V_2.2_HA (green) at (top to bottom) DIV 1, 7, 14, 21 and 28. Scale bars for small and medium DRG neurons: 10 µm and 15 µm, respectively. (**B**) and (**C**) Cell surface Ca_V_2.2_HA intensity was measured from all Ca_V_2.2_HA-positive neurons at DIV 1. The mean fluorescence intensity values were then used to normalise cell surface Ca_V_2.2_HA of individual neurons depending on size, in 3 separate experiments, for (**B**) small and (**C**) medium DRG neurons at DIV 1, 7, 14, 21 and 28. Individual data points are shown for a total of 167, 203, 98, 103 and 122 small DRG neurons (**B,** green symbols) and 83, 119, 164, 262 and 359 medium DRG neurons (**C**, blue symbols), respectively. The mean for each experiment is shown in larger symbols and mean ± SEM (n=3) is superimposed. Statistical analysis: one-way ANOVA with Dunnett’s post-hoc test for multiple comparisons to DIV 1. For B: DIV 1 vs DIV 15, ** p = 0.0033; DIV 1 vs DIV 22, * p = 0.0101. For C: DIV 1 vs DIV 15, * p = 0.0171.

**
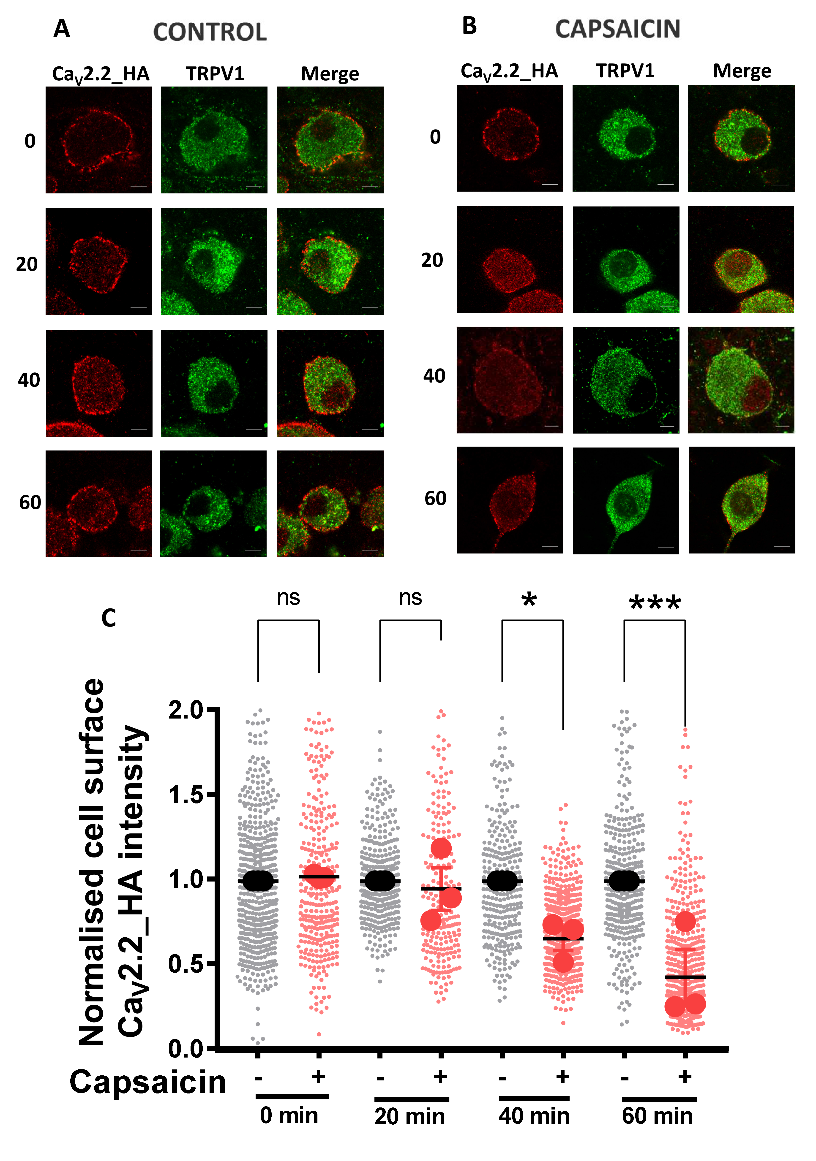
**

**Supplementary Figure 4: Decrease in cell surface Ca_V_2.2_HA in small DRG neurons at 40 and 60 min after application of capsaicin**

Capsaicin or control medium (2 min) was applied to DIV 21 co-cultures, followed by rest for 0, 20, 40 and 60 min at 37^o^C prior to fixation. Representative images of small DRG neurons from control (**A**) and capsaicin (**B**) conditions. Ca_V_2.2_HA (left, red), TRPV1 (middle, green) and merged image (right), after rest for (top to bottom) and 0, 20, 40 and 60 min. Scale bar: 5 µm. (**C**) Normalized cell surface Ca_V_2.2_HA intensity following control or capsaicin application and rest for 0, 20, 40 and 60 min. For analysis, cell surface Ca_V_2.2_HA fluorescence intensity was measured from all TRPV1-positive neurons and the perimeter of these DRG neurons was used as an estimation of neuron size, the size-groups were analysed independently. In all experiments, the fluorescence intensity of each neuron was normalised against the mean fluorescence of control DRG neurons from their respective size-groups. Individual data points represent normalized Ca_V_2.2_HA intensity measured from three separate experiments and a total of 554, 354, 297 and 357 (control, grey circles) and 317, 249, 542 and 548 (capsaicin, red circles), respectively, normalised to control timepoint 0 min. Mean values for each experiment are shown by larger symbols, and mean ± SEM (n=3) is superimposed. Statistical analysis: one-way ANOVA with Sidak’s selected multiple comparisons post-hoc test; * p = 0.0264; ***p = 0.0004; ns, not significant.

**
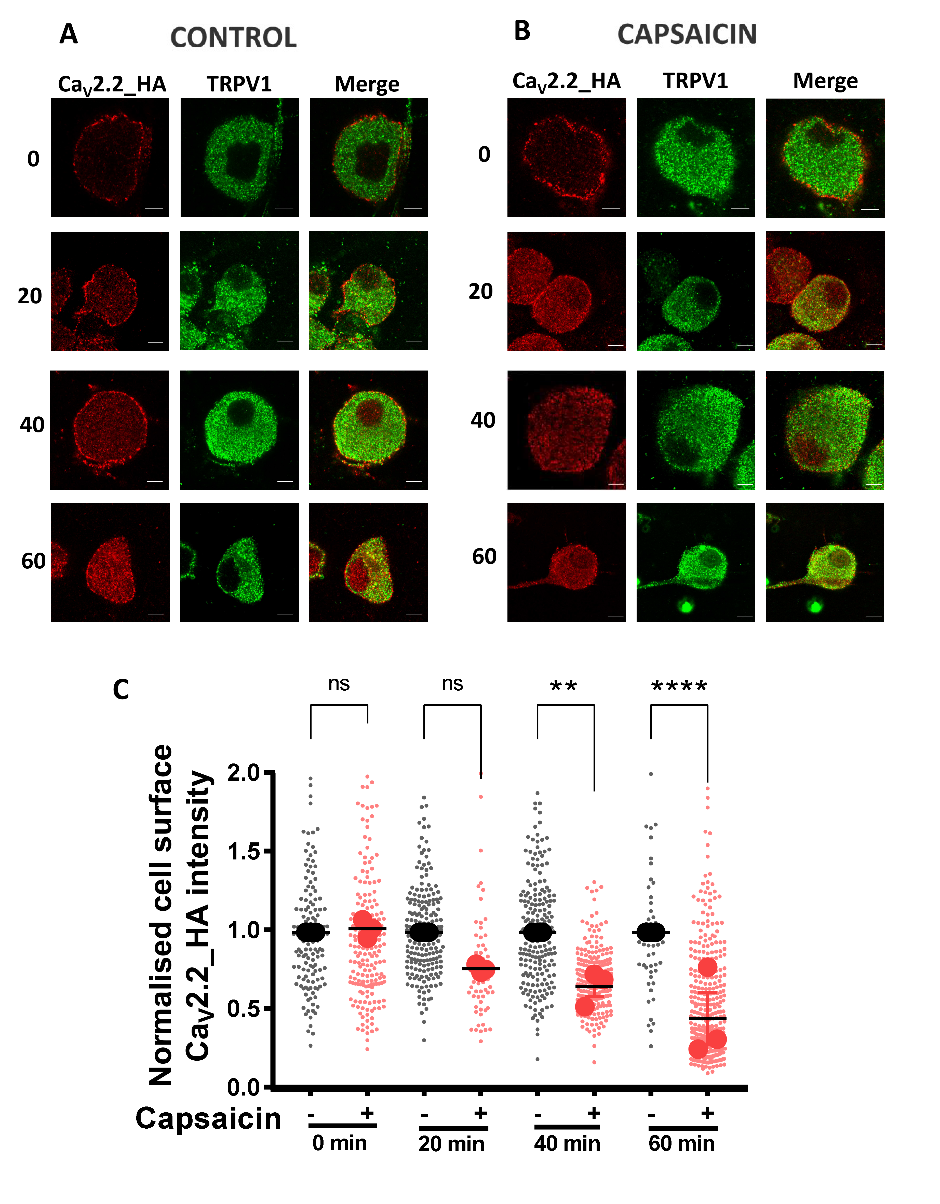
**

**Supplementary Figure 5: Decrease in cell surface Ca_V_2.2_HA in medium DRG neurons at 40 and 60 min after application of capsaicin**

Capsaicin or control medium (2 min) was applied to DIV 21 co-cultures, followed by rest for 0, 20, 40 and 60 min at 37^o^C prior to fixation. Representative images of medium DRG neurons from control (**A**) and capsaicin (**B**) conditions. Ca_V_2.2_HA (left, red), TRPV1 (middle, green) and merged image (right), after rest for (top to bottom) and 0, 20, 40 and 60 min. Scale bar: 5 µm. (**C**) Analysis was performed as described in the legend to Supplementary Fig. 5. Normalized cell surface Ca_V_2.2_HA intensity following control or capsaicin application and rest for 0, 20, 40 and 60 min. Individual data points represent normalized Ca_V_2.2_HA intensity measured from three separate experiments, and a total of 140, 209, 210 and 50 (control, grey circles) and 199, 64, 229 and 343 (capsaicin, red circles), respectively, normalised to control timepoint 0 min. . Mean values for each experiment are shown by larger symbols, and mean ± SEM (n=3) is superimposed. Statistical analysis: one-way ANOVA with Sidak’s multiple comparisons post-hoc test; ** p = 0.0057; **** p<0.0001; ns, not significant.

**
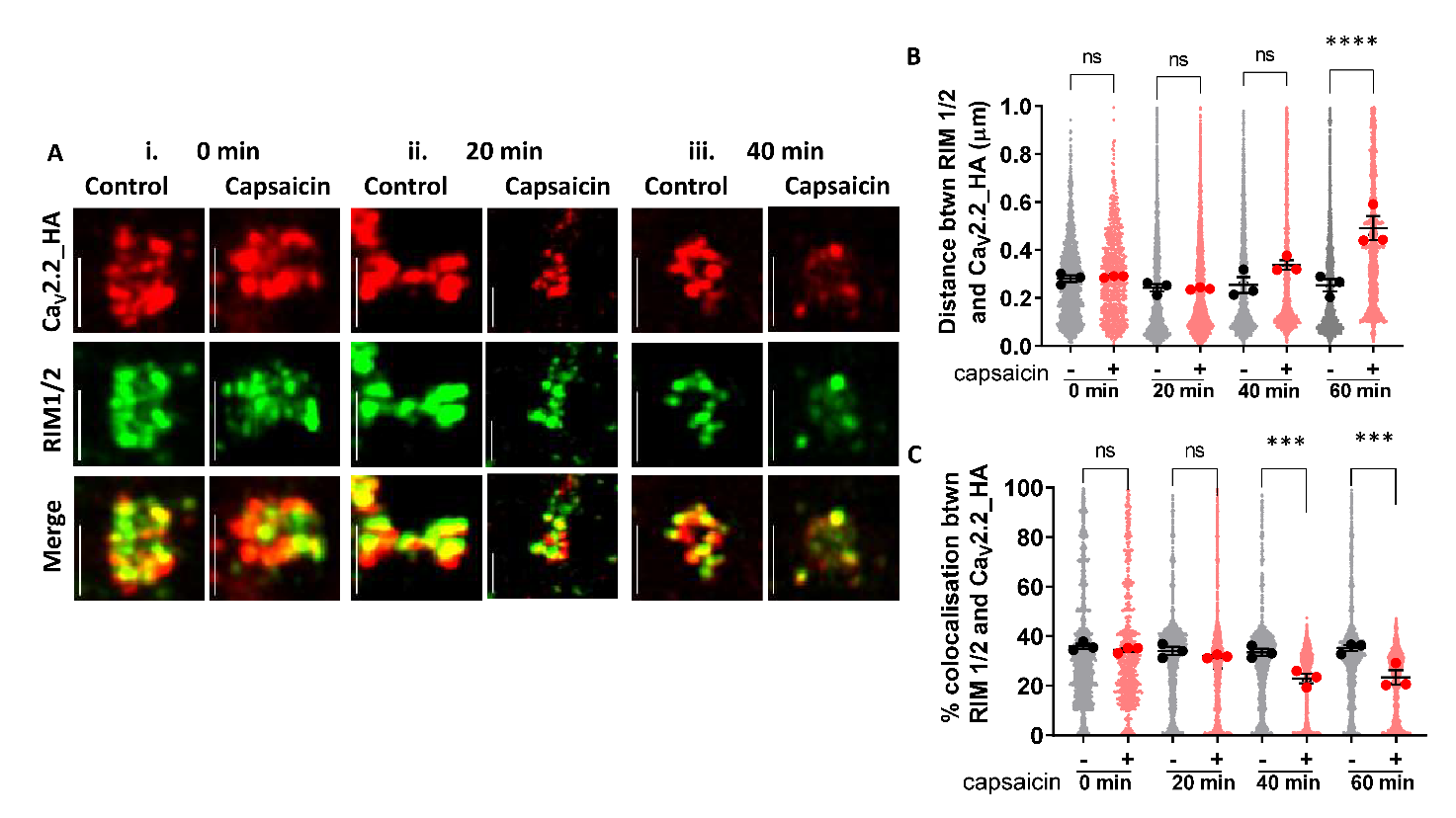
**

**Supplementary Figure 6: Ca_V_2.2_HA co-localization with RIM 1/2 is reduced with a slow time course in presynaptic terminals of DRG neurons following application of capsaicin**

(**A**) Representative images of DRG presynaptic terminals following capsaicin (2 min application), then rest for (**i**) 0, (**ii**) 20, (**iii**) 40 min at 37^o^C prior to fixation. Ca_V_2.2_HA (red, left), RIM 1/2 (green, middle) and merged image (right) in control and capsaicin conditions. Scale bars: 2 µm. (**B**) Distance measurements between centres of co-localized objects Ca_V_2.2_HA and RIM 1/2. (**C**) Measurements of the percentage co-localizing volume for each object’s pair for Ca_V_2.2_HA and RIM 1/2. For (**B**) and (**C**), individual data points represent distance and percentage measurements between co-localizing objects for 2300 pairs, for both control (black) and capsaicin (red) conditions. Mean data for each of 3 experiments is shown in larger symbols and mean ± SEM (n = 3) is superimposed. Statistical analysis: one-way ANOVA with Sidak’s multiple comparisons post-hoc test for the different time points. For (B) at 60 min, **** p < 0.0001. For (C) at 40 min, *** p = 0.0009; at 60 min, *** p = 0.0003. ns = not significant.

**
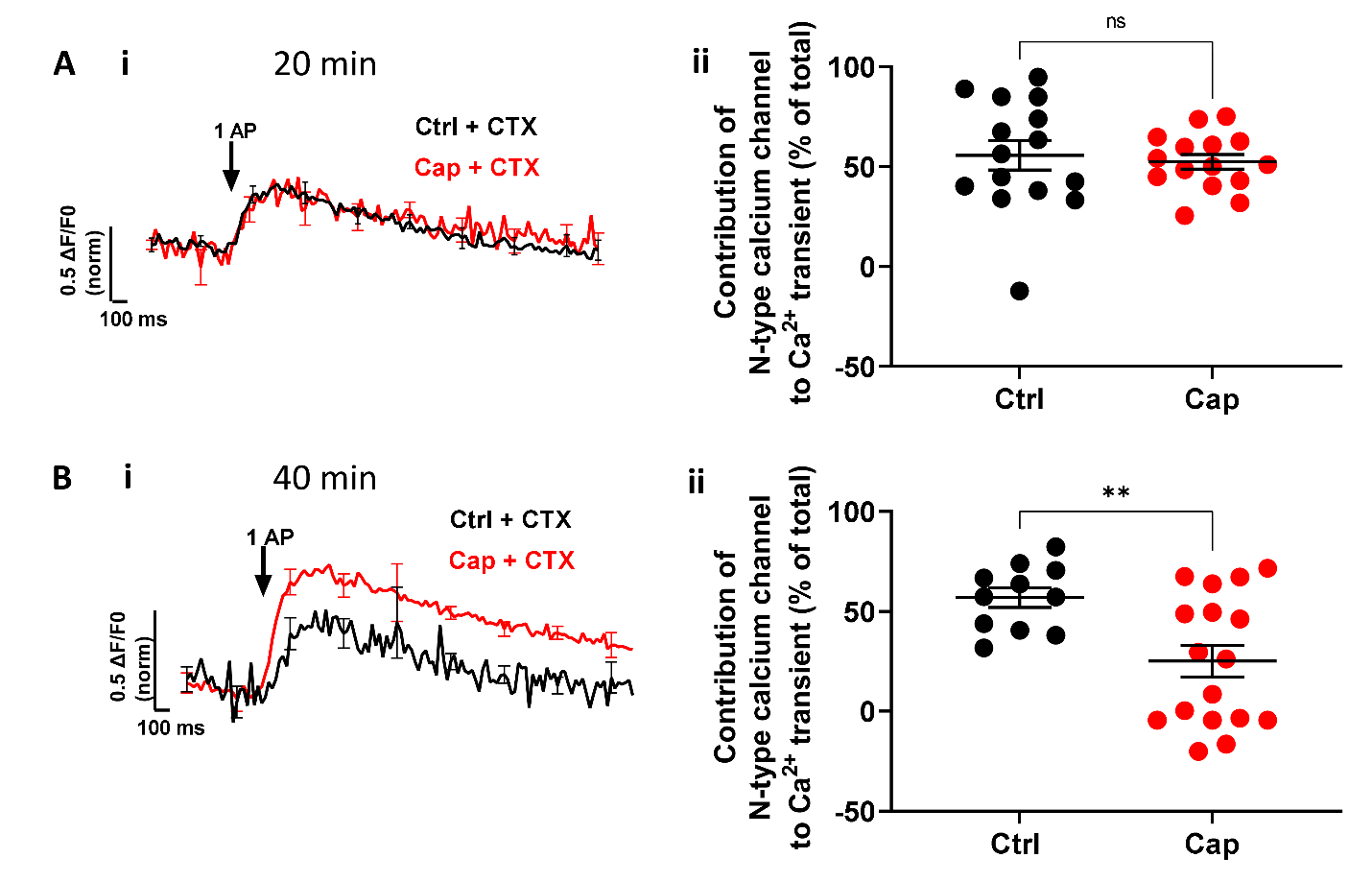
**

**Supplementary Figure 7: Capsaicin application reduces N-type VGCC contribution to 1 AP Ca^2+^ transients following 20 or 40 min rest.**

(**A i**) and (**B i**) Average normalised Sy-GCaMP6f fluorescence change in response to 1 AP stimulation recorded from VAMP positive DRG synaptic terminals of capsaicin (2 min)-treated or control DIV 21 co-cultures, followed by (**A**) 20 or (**B**) 40 min rest. Control medium (black trace) or capsaicin (Cap; red trace), followed by rest for time stated, then CTX application (according to protocol in Fig. 6A). (**A ii**) and (**B ii**) CTX was used to determine the N-type VGCC contribution to 1 AP stimulation. The 1 AP Ca^2+^ transient is expressed as ΔF/F0 and normalised to the averaged peak recorded from VAMP-positive boutons before control medium or capsaicin and CTX was applied. For (**A ii**) control (black circles) n = 15 and Capcaisin (red circles) n = 15. For (B ii) control (black circles) n = 11 and capsaicin (red circles) n = 17. Mean ± SEM are superimposed. Statistical analysis: Mann-Whitney test; *p = 0.0022, ns = not significant.

**
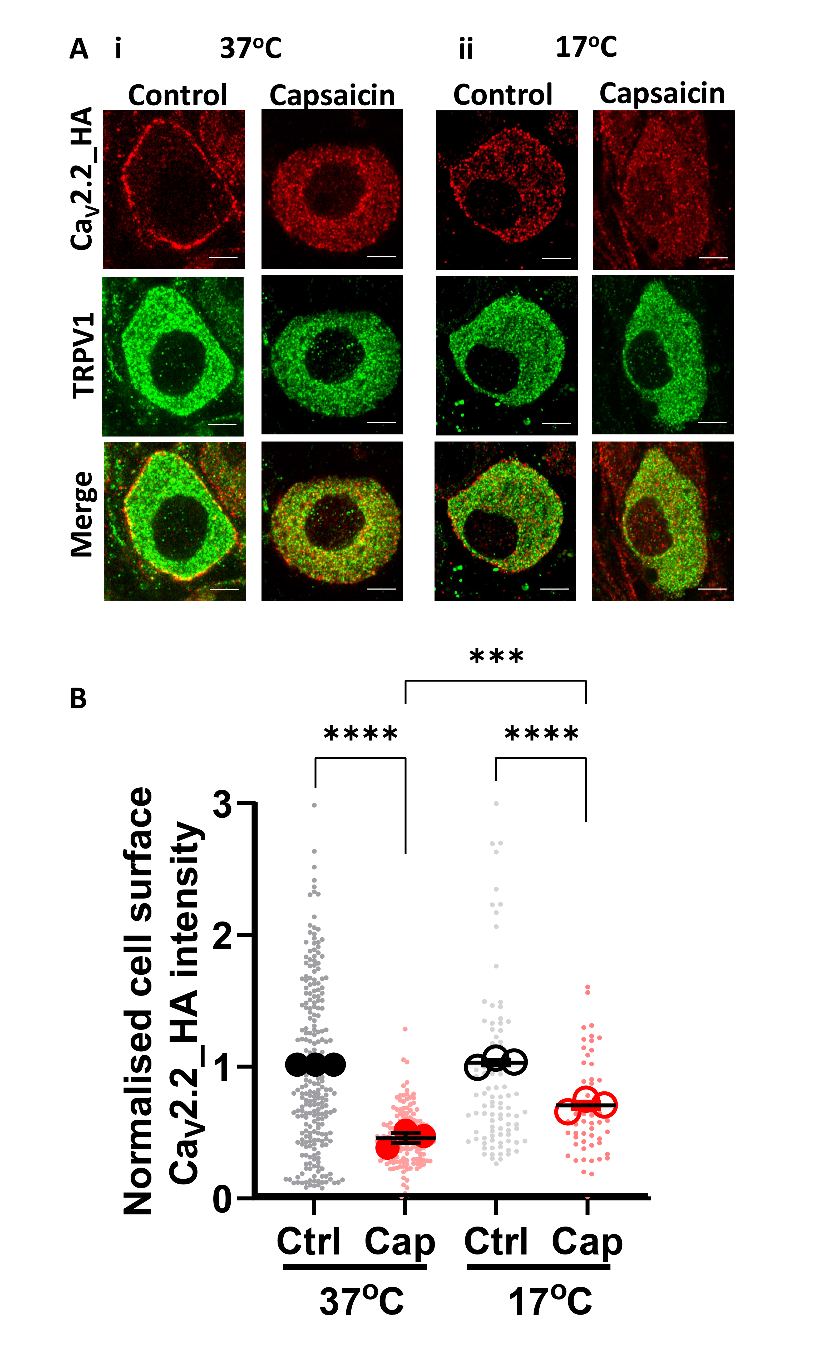
**

**Supplementary Figure 8: Decrease in cell surface Ca_V_2.2_HA in capsaicin-treated medium DRG neurons following rest at 17^o^C**

(**A**) Capsaicin or control medium (2 min at 37^o^C) application to co-cultures (DIV 21), was followed by rest for 60 min at (**i**) 37^o^C or (**ii**) 17^o^C, prior to fixation. Representative images from medium DRG neurons showing Ca_V_2.2_HA (top, red), TRPV1 (middle, green) and merged image (bottom). Scale bars: 5 µm. (**B)** Ca_V_2.2_HA intensity (individual data points are normalized to mean value of medium DRG neurons in control conditions at 37^o^C). Individual data points measured from three separate experiments and a total of 235 and 154 cells for control and capsaicin conditions at 37^o^C (grey and red solid circles, respectively) and 101 and 54 from control and capsaicin conditions at 17^o^C (grey and red open circles, respectively). Mean values for each experiment are shown by larger back and red symbols. Mean ± SEM (n=3) is superimposed. Statistical analysis: one-way ANOVA with Sidak’s multiple comparisons post-hoc test; ***p = 0.0004 and ****p<0.0001.
